# Synthesis of Disaccharide Nucleosides Utilizing the Temporary Protection of the 2′,3′-*cis*-Diol of Ribonucleosides by a Boronic Ester

**DOI:** 10.3390/molecules22101650

**Published:** 2017-10-01

**Authors:** Hidehisa Someya, Taiki Itoh, Shin Aoki

**Affiliations:** 1 Faculty of Pharmaceutical Sciences, Tokyo University of Science, 2641 Yamazaki, Noda, Chiba 278-8510, Japan; 3b17705@ed.tus.ac.jp (H.S.); 3b16703@ed.tus.ac.jp (T.I.); 2 Imaging Frontier Center, Tokyo University of Science, 2641 Yamazaki, Noda, Chiba 278-8510, Japan

**Keywords:** disaccharide nucleoside, glycosylation, boronic ester, temporary protection

## Abstract

Disaccharide nucleosides are an important class of natural compounds that have a variety of biological activities. In this study, we report on the synthesis of disaccharide nucleosides utilizing the temporary protection of the 2′,3′-*cis*-diol of ribonucleosides, such as adenosine, guanosine, uridine, 5-metyluridine, 5-fluorouridine and cytidine, by a boronic ester. The temporary protection of the above ribonucleosides permits the regioselective *O*-glycosylation of the 5’-hydroxyl group with thioglycosides using a *p*-toluenesulfenyl chloride (*p*-TolSCl)/silver triflate (AgOTf) promoter system to afford the corresponding disaccharide nucleosides in fairly good chemical yields. The formation of a boronic ester prepared from uridine and 4-(trifluoromethyl)phenylboronic acid was examined by ^1^H, ^11^B and ^19^F NMR spectroscopy.

## 1. Introduction

Disaccharide nucleosides, which contain an external sugar moiety linked to one of the hydroxyl groups of the nucleoside via an *O*-glycoside bond, constitute an important class of natural compounds [1,2,3,4,5,6,7]. They are found in biopolymers, such as tRNA and poly(ADP-ribose), as well as antibiotics and other biologically-active compounds [5,6,8,9,10,11]. Adenophostins [12,13,14,15], HF-7 [16], amicetin analogs [6,17], ezomycin [18] and some candidates for inhibitors of chitin synthase [19] are typical examples of disaccharide nucleosides that contain adenine, guanine, cytosine and uracil moieties, respectively. Therefore, disaccharide nucleosides and their analogs would be expected to be good drug candidates.

Several strategies for the synthesis of disaccharide nucleosides such as enzymatic *O*-glycosylation [20,21], chemical *N*-glycosylation [5,9,16,22,23,24] and chemical *O*-glycosylation [7,9,14,16,18,19,24,25,26,27,28,29,30,31,32,33,34,35,36,37] have been reported to date. Chemical *O*-glycosylation is often useful for the large-scale synthesis of the desired disaccharide nucleosides in higher chemical yields compared to chemical *N*-glycosylation. However, the neutralization of promoters, which are generally Lewis or Brønsted acids, by the nucleobase moieties would be a possible drawback. Moreover, it is reported that an excess amount of the glycosyl donor is required for glycosylation at the hydroxyl site to be complete, because it is likely that glycosylation preferentially proceeds on the nucleobase or other Lewis basic site [18,32,34,36]. Side reactions such as depurination (cleavage of the anomeric C–N bond of nucleosides), anomerization reaction and trans-purinylation have also been reported [35,38,39].

**Figure 1:**
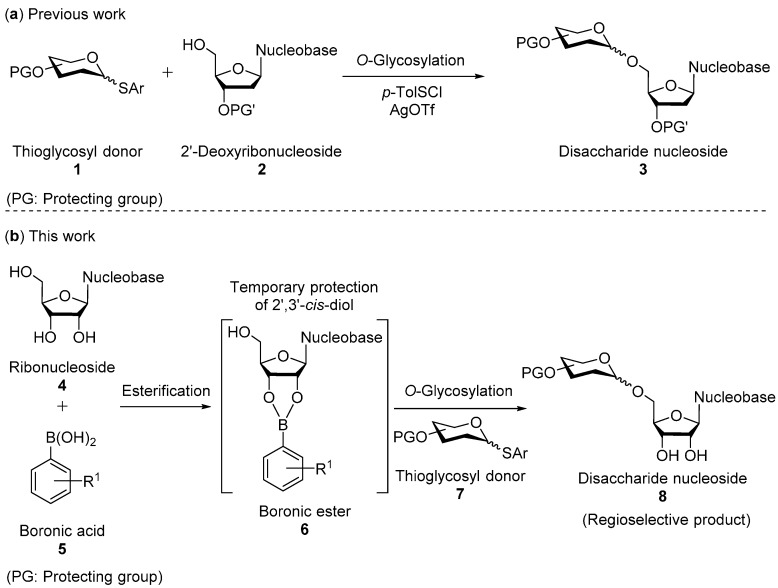
(**a**) *O*-glycosylation of 2′-deoxyribonucleoside with a thioglycosyl donor using the *p*-toluenesulfenyl chloride (*p*-TolSCl)/silver triflate (AgOTf) promoter system; (**b**) regioselective *O*-glycosylation of ribonucleoside at the 5′-OH position via temporary protection of 2′,3′-*cis*-diol.

We previously reported on the synthesis of disaccharide nucleosides **3** by the direct *O*-glycosylation of 2’-deoxyribonucleoside **2** with the thioglycosyl donor **1** (PG: protecting group) (Figure 1a) [40]. Among the glycosyl promoters tested, a combination of *p*-toluenesulfenyl chloride (*p*-TolSCl) and silver triflate (AgOTf) was found to give the corresponding products in moderate to high chemical yields. These results prompted us to investigate the synthesis of disaccharide nucleosides via the *O*-glycosylation of ribonucleosides. The synthesis of disaccharide nucleosides using protected ribonucleosides as glycosyl acceptors, which requires tedious protecting group manipulations, has been reported in previous studies [7,9,14,16,18,19,24,32,33,34,35,36,37]. The development of direct and regioselective *O*-glycosylation using unprotected or temporarily-protected ribonucleosides would afford a more convenient synthetic route to prepare various biologically-active derivatives.

In this manuscript, we report on the *O*-glycosylation of unprotected ribonucleosides **4** at the 5′-hydroxyl group via the temporary protection of the 2′,3′-*cis*-diol by a boronic ester **6**. It has been reported that boronic and borinic acids are capable of forming the cyclic esters with carbohydrate derivatives [41,42], and such derivatives have been utilized for regio- and/or stereo-selective alkylation, acylation, silylation and glycosylation [43,44,45,46,47,48,49,50,51,52,53]. In our strategy, the ribonucleoside **4** is treated with the boronic acid **5** to temporarily protect the 2′,3′-*cis*-diol of **4** to prepare **6** in situ, which is then *O*-glycosylated at the 5‘-hydroxyl group with the glycosyl donor **7** to afford the disaccharide nucleosides **8** in a regioselective manner (Figure 1b) (in this manuscript, “disaccharide nucleosides” include the glycosylated deoxyribonucleosides and ribonucleosides, due to the generally-used terminology).

## 2. Results and Discussion

### 2.1. O-Glycosylation of Nucleosides with Thioglycosyl Donors

We first examined the *O*-glycosylation of uridine **10** with the thiomannoside **9** [54] using 3.0 equivalents of *p*-TolSCl and 6.0 equivalents of AgOTf [55,56] against **10** (i.e., 2.0 equivalents of *p*-TolSCl and 4.0 equivalents of AgOTf against **9** according to our previous paper [40]). Thioglycosides are one of the most popular glycosyl donor due to their ease of preparation and modification, high stability and the many available activation methods [25,26,27,28,29,57]. After the glycosylation and crude purification, the resulting compounds were acetylated to permit the desired products to be purified more easily.

The results for the glycosylation reactions are summarized in Table 1. In Entry 1, the glycosylation of **10** with **9** without boronic acid derivatives gave a complex mixture. In Entry 2, a mixture of **10** and phenylboronic acid **11a** was co-evaporated with pyridine and 1,4-dioxane followed by stirring in 1,4-dioxane under reflux conditions [44] to prepare the temporary 2′,3′-*cis*-diol-protected intermediate **6** (in Figure 1), to which **9** (corresponding to **7** in Figure 1) was added. The glycosylation of **6** proceeded at its 5′-OH to afford **12** (corresponding to **8** in Figure 1) in 41% (α/β = 1.6/1) in a regioselective manner. The formation of a 1′′,5′-glycosidic linkage of **12** was confirmed by comparing its ^1^H NMR spectrum with that of the authentic sample prepared by another synthetic route, in which the chemical yield was 20% for four steps from **10** to **12** (excluding the steps required for the preparation of **9**; see Scheme S1 in the Supplementary Materials). In Entry 3, a mixture of **9**, **10** and **11a** was co-evaporated with pyridine and 1,4-dioxane, and the resulting mixture was treated with promoters to give **12** in a yield nearly similar to that for Entry 2. In the following Entries 4–10, therefore, glycosylation reactions were conducted using a procedure similar to that used in Entry 3 for easy manipulation.

The electrostatic effect of the substituents of the boronic acid was studied in Entries 4–6. Glycosylations using 4-methoxyphenylboronic acid (4-MeOPhB(OH)_2_) **11b**, 4-(trifluoromethyl)phenylboronic acid (4-CF_3_PhB(OH)_2_) **11c** and 2,4-difluorophenylboronic acid (2,4-F_2_PhB(OH)_2_) **11d** were conducted to give **12** in 39%, 51% and 46%, respectively, suggesting the positive effect of electron-withdrawing moieties such as -CF_3_ and -F on the aromatic ring of the boronic acid.

The solvent effect was also examined in Entries 7–9. It is well known that glycosylation in an ether-type solvent such as Et_2_O, THF and 1,4-dioxane enhances α-stereoselectivity [58,59]. As shown in Entry 7, 1,4-dioxane improved the α-stereoselectivity of the reaction, while the chemical yield was unsatisfactory. In Entry 8, CH_2_Cl_2_ gave a negligible amount of **12**, due to the low solubility of the substrates. Glycosylation using EtCN gave **12** in higher chemical yield (Entry 9) than those for 1,4-dioxane (Entry 7) and MeCN (Entry 3), and the stereoselectivity was nearly the same as that in MeCN (Entry 3).

In Entry 10, glycosylation using lower equivalents of promoters (1.8 equivalents of *p*-TolSCl and 3.6 equivalents of AgOTf against **10**) than those in Entry 9 gave similar results. Therefore, 3.0 equivalents and 6.0 equivalents of *p*-TolSCl and AgOTf were used in the following *O*-glycosylations to complete the reactions. In Entry 11, phenylboronic acid having a C6 alkyl chain **11e** was used to improve the solubility of the boronic ester, albeit the chemical yield was not improved.

The *O*-glycosylation of adenosine **13** with **9** was examined next. As shown in Entry 1 of Table 2, *O*-glycosylation in the absence of boronic acid derivatives gave a complex mixture, as in the case of uridine (Entry 1 in Table 1). In Entries 2 and 3, in which PhB(OH)_2_ **11a** and 4-CF_3_PhB(OH)_2_ **11c** were used, **14** was produced, but the yields were lower (14% and 11%, respectively) than those of **10** in Entries 3 and 9 of Table 1, which can be attributed to the trans-purinylation of **13** and/or **14** (*N*-mannosyl adenine **15** was isolated in 6–27%) [36].

We attempted the *O*-glycosylations of various nucleosides **10**, **13** and **16**–**22** with the thiogalactoside **23**, in which the hydroxyl groups were protected by benzoyl groups to achieve β-selective *O*-glycosylation by neighboring group participation at the O2 benzoyl group (Table 3). The formation of a β-1′′,5′-glycosidic linkage between the galactose moiety and ribonucleoside in the products **24**–**32** was confirmed by NNR measurements (^1^H NMR, ^13^C NMR, ^1^H-^1^H COSY, HMQC and HMBC). As listed in Table 3, the reaction of the unprotected and *N*-protected adenosine, **13** and **16** [60], afforded the desired products β-**24** and β-**25** in 42% and 30%, respectively (Entries 1 and 2). Note that the use of unprotected adenosine **13** gave a better yield than that for the protected **16**, phenomena similar to the *O*-glycosylation of 2’-deoxyadenosine reported by us in a previous study (Figure 1a) [40]. It should also be noted that the reaction of **13** with **23** (Entry 1) gave negligible amounts of *N*-galactosyl adenine as a byproduct unlike the use of the mannosyl donor **9** in an *O*-glycosylation reaction (Entries 2 and 3 in Table 2). In Entries 3 and 4, the reaction of the unprotected and *N*-protected guanosine, **17** and **18** [61], afforded the corresponding products β-**26** and β-**27** in 12% and 44%, respectively. The higher yield of β-**27** is possibly due to better solubility of the boronic ester intermediate prepared from the *N*-protected **18** in EtCN than that from the unprotected **17**. In Entry 5, the *O*-glycosylation of uridine **10** with **23** gave the desired product β-**28** in 42% yield, and the electrophilic substitution reaction at the 5-position of the uracil moiety of **10** and/or β-**28** with the *p*-toluenesulfenyl cation was observed (*ca.* 15%) [62]. In Entries 6 and 7, the *O*-glycosylation of 5-metyluridine **19** and 5-fluorouridine **20** afforded the corresponding products β-**29** and β-**30** in 53% and 61%, respectively. In Entries 8 and 9, the reaction of the unprotected and *N*-protected cytidine, **21** and **22** [63], afforded β-**31** and β-**32** in 55% and 40%, respectively. It should be noted that the use of the unprotected **21** gave β-**31** in slightly higher yield than β-**32** from the protected **22**.

The *O*-glycosylation of 5-fluorouridine **20** with glucosyl, galactosyl and mannosyl donors was also examined. As summarized in Table 4, the glucosyl donor **33** [64] and the galactosyl donor **23** afforded the corresponding products β-**35** and β-**30** in reasonably acceptable yields, while the use of the mannosyl donor **34** [65] gave a mixture.

### 2.2. O-Glycosylation of Nucleosides with Thioglycosyl Donors Containing the Boronic Acid Moiety on the Leaving Group

**Figure 2:**
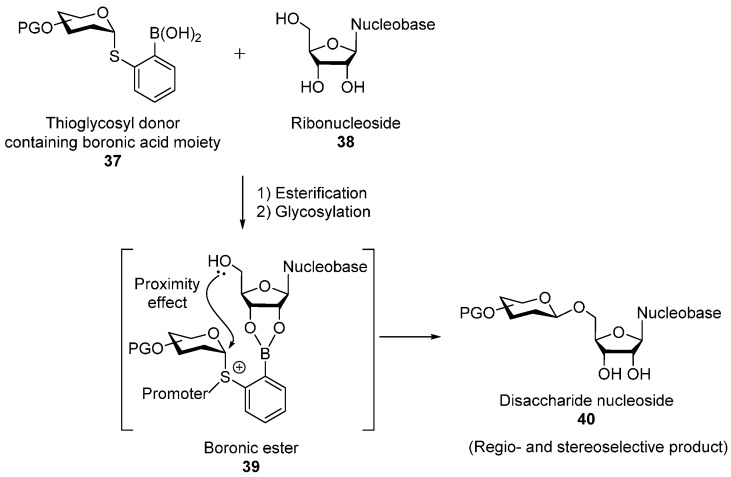
Regio- and stereo-selective *O*-glycosylation of the ribonucleoside utilizing the glycosyl donor containing a boronic acid moiety.

Shen and co-workers recently reported on the 1,2-*cis* glycosylation of some simple alcohols using glucosyl donors containing a boronic acid moiety on the leaving group, which is referred to leaving group-based aglycon delivery [48]. These results prompted us to examine the use of the thioglycosyl donor **37** containing a boronic acid moiety on the leaving group, which was expected to form a boronic ester with the 2′,3′-*cis*-diol of ribonucleoside **38** to give the intermediate **39** (Figure 2). It was expected that the *O*-glycosylation of **39** would produce **40** in a pseudo-intramolecular manner.

In Table 5, the results for the *O*-glycosylation of uridine **10** and adenosine **13** with the glycosyl donors **41** and **42** (Schemes S2 and S3 in the Supplementary Materials) are summarized. In Entries 1 and 2, the reactions of **10** and **13** with **41** afforded the corresponding products **12** and **14** in 44% (α/β = 1.9/1) and 16% (α/β = 1.3/1), respectively. In Entries 3 and 4, **42** gave the almost the same chemical yields and stereoselectivities as those in Entries 1 and 2. These results are similar to Entry 3 in Table 1 and Entry 2 in Table 2, in which **10** or **13** was reacted with **9** and phenylboronic acid **11a** under the same conditions in Table 5, indicating that the introduction of a boronic acid on the thiophenyl leaving group in our reactions has a negligible effect on the overall reaction.

### 2.3. Deprotection of the Glycosylation Products

**Scheme 1:**
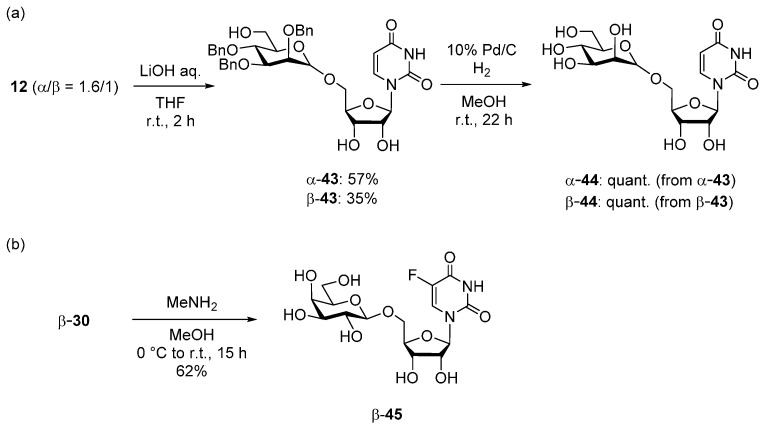
Deprotection of **12** (**a**) and β-**30** (**b**).

The deprotection of the glycosylation product **12** (α/β = 1.6/1) involved a treatment with aqueous LiOH to afford α-**43** and β-**43**, which were separated by silica gel column chromatography. The deprotection of the benzyl groups of α-**43** and β-**43** under traditional reaction conditions (10% Pd/C with H_2_ gas) gave α-**44** and β-**44**, respectively (Scheme 1a) [66]. The deprotection of β-**30** by treatment with MeNH_2_ [67] afforded β-**45** [68] in 62% (Scheme 1b).

### 2.4. Interaction of Uridine and 4-(Trifluoromethyl)phenylboronic Acid Studied by ^1^H, ^11^B and ^19^F NMR Spectroscopy

**Figure 3:**
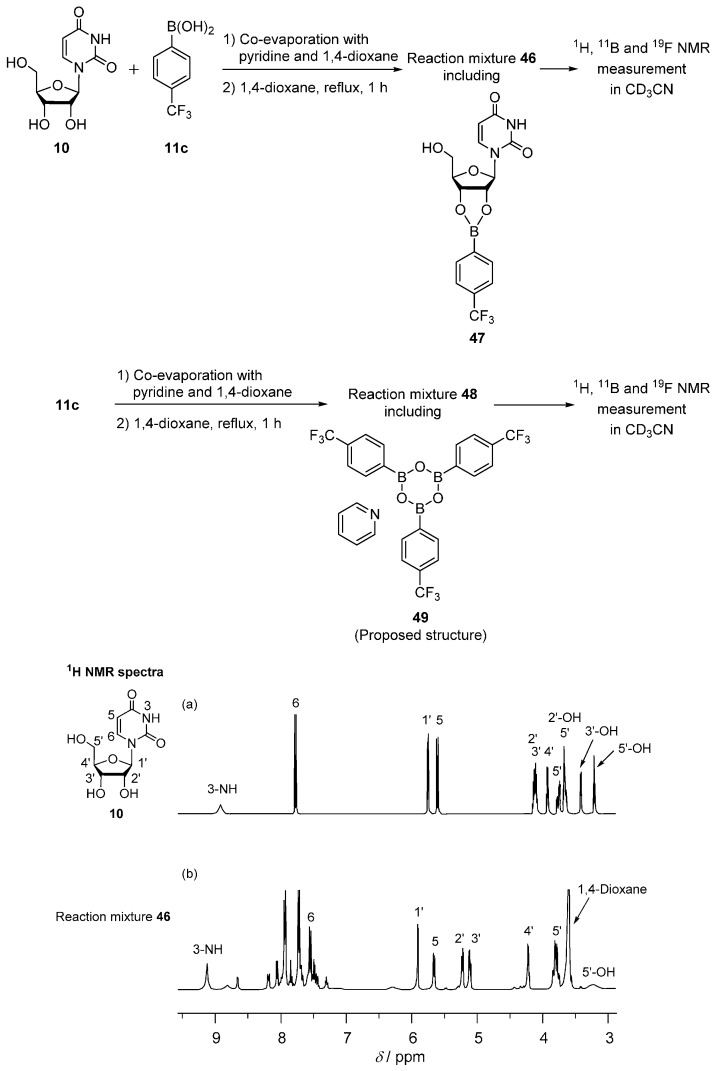

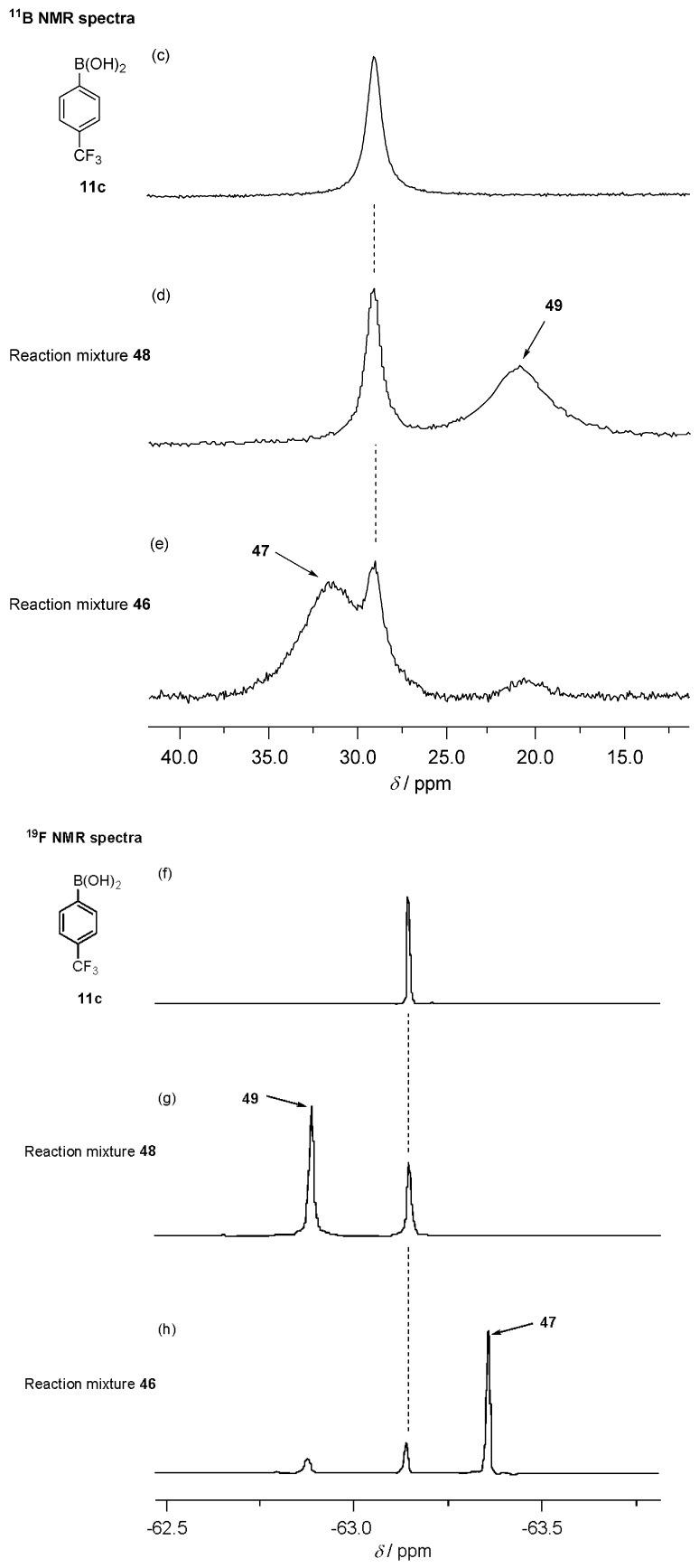
Possible assignment of intermediates from the reaction of uridine **10** and 4-CF_3_PhB(OH)_2_ **11c** in ^1^H, ^11^B and ^19^F NMR spectra (in CD_3_CN at 25 °C). (**a**) ^1^H NMR of **10**; (**b**) ^1^H NMR of a mixture **46**; (**c**) ^11^B NMR of **11c**; (**d**) ^11^B NMR of a mixture **48**; (**e**) ^11^B NMR of a mixture **46**; (**f**) ^19^F NMR of **11c**; (**g**) ^19^F NMR of a mixture **48**; (**h**) ^19^F NMR of a mixture **46**.

The temporary protection of the 2’3’-*cis*-diol of ribonucleoside with a boronic acid was checked by NMR spectroscopy. The ^1^H, ^11^B and ^19^F NMR measurements of uridine **10**, 4-(trifluoromethyl)phenylboronic acid **11c** and a mixture of **10** and **11c** were undertaken in CD_3_CN (Figure 3). For the preparation of the third sample, a mixture of **10** and **11c** was azeotroped with pyridine and 1,4-dioxane, followed by stirring in 1,4-dioxane under the reflux conditions for 1 h. For comparison, **11c** was azeotroped in a similar manner, and the ^11^B and ^19^F NMR spectra of the resulting mixture were obtained. As shown in Figure 3a,b, the peaks for the 2′ and 3′ hydroxyl groups disappeared, and the 2′ and 3′ proton signals were shifted considerably upfield upon the addition of **11c**. In Figure 3c–e, it was assumed that the peaks at 21 ppm, 28 ppm and 32 ppm correspond to a 2,4,6-tris[4-(trifluorometyl)phenyl]boroxine pyridine complex, the proposed structure of which is **49** (some NMR spectra of boroxine pyridine complexes were reported [69,70,71]), **11c** or 2,4,6-tris[4-(trifluorometyl)phenyl]boroxine and the desired boronic ester **47**, respectively. In Figure 3f–h, we assumed that the peaks at −63.3 ppm, −63.2 ppm and −62.8 ppm correspond to **47**, **11c** or 2,4,6-tris[4-(trifluorometyl)phenyl]boroxine and **49**, respectively.

## 3. Materials and Methods

### 3.1. General Information

Reagents and solvents were commercially purchased, were the highest commercial quality available and were used without further purification. Anhydrous CH_2_Cl_2_ was prepared by distillation from calcium hydride. Acetonitrile and propionitrile were prepared by distillation from calcium hydride and the successive distillation from phosphorus (V) oxide. Anhydrous 1,4-dioxane was prepared by distillation from sodium. All aqueous solutions were prepared using deionized water.

^1^H (300 and 400 MHz), ^11^B (128 MHz), ^13^C (75 and 100 MHz) and ^19^F (376 MHz) NMR spectra were recorded on a JEOL Always 300 (JEOL, Tokyo, Japan) and a JEOL Lamda 400 (JEOL, Tokyo, Japan) spectrometer. Tetramethylsilane (TMS) was used as an internal reference for ^1^H and ^13^C NMR measurements in CDCl_3_, CD_3_OD, CD_3_CN, acetone-*d*_6_ and DMSO-*d*_6_. 3-(Trimethylsilyl)propionic-2,2,3,3-*d*_4_ acid sodium (TSP) was used as an internal reference for ^1^H NMR measurements in D_2_O. 1,4-Dioxane was used as an internal reference for ^13^C NMR measurements in D_2_O. ^11^B and ^19^F NMR spectra were measured in a quartz NMR tube. The boron trifluoride-diethyl ether complex (BF_3_·OEt_2_) in CDCl_3_ was used as an external reference (0 ppm) for ^11^B NMR, and trifluoroacetic acid (TFA) in CDCl_3_ was used as an external reference (−76.5 ppm) for ^19^F NMR. IR spectra were recorded on a Perkin-Elmer FTIR Spectrum 100 (ATR) (PerkinElmer, Massachusetts, USA). MS measurements were performed on a JEOL JMS-700 (JEOL, Tokyo, Japan) and Varian 910-MS (Varian Medical Systems, California, USA) spectrometer. Elemental analyses were performed on a Perkin-Elmer CHN 2400 analyzer (PerkinElmer, Massachusetts, USA). Optical rotations were measured with a JASCO P-1030 digital polarimeter (JASCO, Tokyo, Japan) in 50-mm cells using the D line of sodium (589 nm). Thin-layer chromatography (TLC) and silica gel column chromatography were performed using Merck Silica gel 60 F_254_ plate (Merck KGaA, Darmstadt, Germany) and Fuji Silica Chemical FL-100D (Fuji Silysia Chemical, Aichi, Japan), respectively. HPLC experiments were carried out using a system consisting of a PU-2089 Plus intelligent HPLC pump (JASCO, Tokyo, Japan), a UV-2075 Plus intelligent UV-visible detector (JASCO, Tokyo, Japan), a Rheodine injector (Model No. 7125) and a Chromatopak C-R8A (Shimadzu, Kyōto, Japan). For preparative HPLC, a SenshuPak Pegasil ODS column (Senshu Scientific Co., Ltd., Tokyo, Japan) (20φ × 250 mm, No. 0509271H) was used. GPC experiments were carried out using a system consisting of a POMP P-50 (Japan Analytical Industry Co., Ltd., Tokyo, Japan), a UV/VIS DETECTOR S-3740 (Soma, Tokyo, Japan), a Manual Sample Injector 7725i (Rheodyne, Bensheim, Germany) and an MDL-101 1 PEN RECORDER (Japan Analyrical Industry Co., Ltd., Tokyo, Japan), equipped with two GPC columns, JAIGEL-1H and JAIGEL-2H (Japan Analyrical Industry Co., Ltd., Tokyo, Japan) (20φ × 600 mm, No. A605201 and A605204).

### 3.2. Synthesis of Compounds

*2′,3′-Di-O-acetyl-5′-O-(6′′-O-acetyl-2′′,3′′,4′′-tri-O-benzyl-α/β-d-mannopyranosyl)uridine* (**12**) (Entry 9 in Table 1): A mixture of **9** (28.4 mg, 48.6 µmol), **10** (7.9 mg, 32.4 µmol) and **11c** (9.3 mg, 49.0 µmol) was co-evaporated with anhydrous pyridine (three times) and anhydrous 1,4-dioxane (three times) and dissolved in anhydrous 1,4-dioxane (320 µL). This reaction mixture was stirred under reflux conditions for 1 h and concentrated under reduced pressure. The resulting mixture was stirred with activated 4 Å molecular sieves (64 mg) in anhydrous EtCN (640 µL) at room temperature for 30 min and then cooled to −40 °C, to which *p*-TolSCl (12.8 µL, 96.8 µmol) and AgOTf (49.9 mg, 194 µmol) were added at the same temperature. After stirring for 1.5 h at −40 °C, the reaction mixture was quenched with saturated aqueous NaHCO_3_, diluted with CHCl_3_ and filtered through Celite. The organic layer was washed with saturated aqueous NaHCO_3_ and brine, dried over Na_2_SO_4_, filtered and concentrated under reduced pressure. The remaining residue was purified by silica gel column chromatography (CHCl_3_/MeOH = 1/0–50/1) to give 5′-*O*-(6′′-*O*-acetyl-2′′,3′′,4′′-tri-*O*-benzyl-α/β-d-mannopyranosyl)uridine including a small amount of byproducts as a colorless syrup (15.2 mg). To the resulting crude compound in anhydrous pyridine (200 µL), Ac_2_O (20.4 µL, 21.6 µmol, 10.0 equiv. based on the crude compound) and DMAP (catalytic amount) were added at 0 °C. The reaction mixture was stirred at the same temperature for 30 min and then allowed to warm to room temperature. After stirring overnight, the reaction mixture was diluted with CHCl_3_, washed with 1 M aqueous HCl, saturated aqueous NaHCO_3_ and brine, dried over Na_2_SO_4_, filtered and concentrated under reduced pressure. The residue was purified by silica gel column chromatography (CHCl_3_/MeOH = 1/0–90/1) to give **12** as a colorless amorphous solid (15.8 mg, 61% yield for 3 steps, α/β = 1.6/1): ^1^H NMR (300 MHz, CDCl_3_, TMS): δ = 8.56 (s, 0.6H), 8.29 (s, 0.4H), 7.89 (d, *J* = 8.1 Hz, 0.4H), 7.41–7.19 (m, 15.6H), 6.29 (d, *J* = 7.2 Hz, 0.4H), 6.15–6.05 (m, 0.6H), 5.55 (dd, *J* = 5.1, 1.2 Hz, 0.4H), 5.39 (dd, *J* = 8.1, 1.8 Hz, 0.6H), 5.33–5.23 (m, 2H), 5.01–4.86 (m, 2H), 4.80–4.55 (m, 4.6H), 4.46 (s, 0.4H), 4.39–4.21 (m, 3H), 4.13 (dd, *J* = 10.5, 1.8 Hz, 0.4H), 4.05 (d, *J* = 2.7 Hz, 0.4H), 3.99–3.84 (m, 2.2H), 3.84–3.68 (m, 1.6H), 3.68–3.57 (m, 1H), 3.44 (dt, *J* = 9.9, 6.9 Hz, 0.4H), 2.15 (s, 1.2H), 2.12 (s, 1.8H), 2.09 (s, 1.2H), 2.09 (s, 1.8H), 2.06 (s, 1.8H), 2.00 (s, 1.2H) ppm; ^13^C NMR (100 MHz, CDCl_3_, TMS): δ = 170.9, 170.8, 170.1, 169.8, 169.7, 169.6, 162.8, 162.6, 150.8, 150.4, 141.0, 138.8, 138.2, 137.9, 137.8, 137.7, 128.5, 128.5, 128.4, 128.4, 128.1, 128.1, 128.0, 127.9, 127.8, 127.7, 127.6, 103.3, 103.2, 100.0 (C_1′′_, ^1^*J*_CH_ = 153.6 Hz, β form), 98.5 (C_1′′_, ^1^*J*_CH_ = 171.0 Hz, α form), 86.3, 85.2, 82.8, 82.1, 81.4, 80.1, 77.3, 75.2, 75.2, 75.0, 74.8, 74.7, 74.3, 73.9, 73.7, 73.5, 72.9, 72.8, 72.5, 72.2, 71.9, 71.2, 71.0, 68.6, 66.8, 63.3, 63.2, 20.9, 20.9, 20.7, 20.6, 20.4 ppm; IR (ATR): *ν* = 3200, 3065, 3032, 2930, 2877, 1742, 1691, 1498, 1455, 1373, 1310, 1231, 1073, 1042, 1029, 925, 901, 811, 737, 697, 635, 597 cm^−1^; HRMS (FAB+): calcd. for [M + H]^+^, C_42_H_47_N_2_O_14_, 803.3027; found, 803.3028.

*2′,3′-Di-O-acetyl-5′-O-(6′′-O-acetyl-2′′,3′′,4′′-tri-O-benzyl-α/β-d-mannopyranosyl)adenosine* (**14**) and *7-N-(6′-O-acetyl-2′,3′,4′-tri-O-benzyl-α-d-mannopyranosyl)adenine* (**15**) (Entry 2 in Table 2): A mixture of **9** (28.4 mg, 48.6 µmol), **13** (8.6 mg, 32.2 µmol) and **11a** (5.9 mg, 48.4 µmol) was co-evaporated with anhydrous pyridine (three times) and anhydrous 1,4-dioxane (three times) and dissolved in anhydrous 1,4-dioxane (320 µL). This reaction mixture was stirred under reflux conditions for 1 h and concentrated under reduced pressure. The resulting mixture was stirred with activated 3 Å molecular sieves (64 mg) in anhydrous MeCN (640 µL) at room temperature for 30 min and then cooled to −20 °C, to which *p*-TolSCl (12.8 µL, 96.8 µmol) and AgOTf (49.9 mg, 194 µmol) were added at the same temperature. After stirring for 1.5 h at −20 °C, the reaction mixture was quenched with saturated aqueous NaHCO_3_, diluted with CHCl_3_ and filtered through Celite. The organic layer was washed with saturated aqueous NaHCO_3_ and brine, dried over Na_2_SO_4_, filtered and concentrated under reduced pressure. The resulting residue was purified by silica gel column chromatography (CHCl_3_/MeOH = 1/0–10/1) to give 5′-*O*-(6′′-*O*-acetyl-2′′,3′′,4′′-tri-*O*-benzyl-α/β-d-mannopyranosyl)adenosine including a small amount of byproducts as a colorless syrup (6.3 mg). To the resulting crude compound in anhydrous pyridine (200 µL), Ac_2_O (8.0 µL, 84.9 µmol, 10.0 equiv. based on the crude compound) and DMAP (catalytic amount) were added at 0 °C. The reaction mixture was stirred at the same temperature for 30 min and then allowed to warm to room temperature. After stirring overnight, the reaction mixture was diluted with CHCl_3_, washed with aqueous NaHCO_3_ and brine, dried over Na_2_SO_4_, filtered and concentrated under reduced pressure. The residue was purified by silica gel column chromatography (CHCl_3_/MeOH = 1/0–5/1) to give **14** as a colorless amorphous solid (3.8 mg, 14% yield for 3 steps, α/β = 1/1.0) and **15** as a colorless syrup (1.1 mg, 6% yield for 3 steps): **14** (α/β = 1/1.0); ^1^H NMR (300 MHz, CDCl_3_, TMS): δ = 8.36 (s, 1H), 8.35 (s, 0.5H), 7.93 (s, 0.5H), 7.42–7.27 (m, 13.5H), 7.16 (t, *J* = 2.7 Hz, 1.5H), 6.31 (d, *J* = 6.0 Hz, 0.5H), 6.20 (d, *J* = 5.7 Hz,0.5H), 5.92 (t, *J* = 5.7 Hz, 0.5H), 5.78–5.67 (m, 1H), 5.67–5.52 (m, 2.5H), 4.97–4.83 (m, 3H), 4.77–4.45 (m, 5H), 4.45–4.16 (m, 3H), 4.05–3.82 (m, 3H), 3.82–3.62 (m, 1H), 3.58–3.41 (m, 1H), 2.15 (s, 1.5H), 2.13 (s, 1.5H), 2.07 (s, 1.5H), 2.06 (s, 1.5H), 2.03 (s, 1.5H), 2.01 (s, 1.5H) ppm; ^13^C NMR (100 MHz, CDCl_3_, TMS): δ = 171.1, 170.9, 169.9, 169.6, 169.4, 169.3, 155.5, 155.4, 153.4, 153.1, 150.0, 150.0, 139.7, 138.7, 138.4, 138.2, 138.1, 138.0, 137.9, 128.4, 128.4, 128.4, 128.4, 128.4, 128.2, 128.1, 127.9, 127.9, 127.8, 127.7, 127.7, 127.6, 127.6, 127.3, 120.1, 119.8, 100.9 (C_1′′_, ^1^*J*_CH_ = 156.9 Hz, β form), 98.6 (C_1′′_, ^1^*J*_CH_ = 168.5 Hz, α form), 85.7, 85.2, 82.3, 82.2, 81.4, 80.2, 75.3, 75.1, 75.0, 74.5, 74.4, 74.3, 74.1, 73.9, 73.1, 73.0, 72.4, 71.7, 71.1, 70.7, 69.3, 66.6, 63.5, 63.4, 21.0, 20.9, 20.7, 20.6, 20.4, 20.4 ppm; IR (ATR): *ν* = 3332, 3171, 3066, 3032, 2927, 2875, 1742, 1635, 1595, 1498, 1473, 1455, 1424, 1366, 1332, 1293, 1234, 1213, 1071, 1042, 1027, 903, 825, 799, 736, 697, 667, 649, 602 cm^−1^; HRMS (FAB+): calcd. for [M + H]^+^, C_43_H_48_N_5_O_12_, 826.3299; found, 826.3294; **15**; ^1^H NMR (300 MHz, CDCl_3_, TMS): δ = 8.44 (s, 1H), 7.90 (s, 1H), 7.41–7.28 (m, 10H), 7.23–7.19 (m, 1H), 7.18–7.08 (m, 2H), 6.86–6.74 (m, 2H), 5.88 (s, 2H), 5.61 (s, 1H), 4.97 (d, *J* = 10.8 Hz, 1H), 4.77 (s, 2H), 4.70 (d, *J* = 6.3 Hz, 1H), 4.66 (d, *J* = 6.6 Hz, 1H), 4.45 (dd, *J* = 12.0, 3.3 Hz, 1H), 4.26 (dd, *J* = 12.0, 2.4 Hz, 1H), 4.25 (d, *J* = 11.1 Hz, 1H), 4.11 (t, *J* = 9.6 Hz, 1H), 3.96 (s, 1H), 3.88 (dd, *J* = 9.3, 2.7 Hz, 1H), 3.76 (dt, *J* = 9.6, 3.0 Hz, 1H), 1.98 (s, 3H) ppm; ^13^C NMR (75 MHz, CDCl_3_, TMS): δ = 170.0, 160.7, 153.2, 151.6, 143.0, 137.3, 135.9, 128.7, 128.6, 128.5, 128.5, 128.4, 128.3, 128.3, 128.2, 127.7, 111.8, 85.7, 82.8, 76.7, 76.1, 75.4, 75.1, 73.0, 72.5, 62.2, 20.7 ppm; IR (ATR): *ν* = 3449, 3371, 3167, 3089, 3064, 3031, 2927, 2873, 1742, 1627, 1587, 1551, 1497, 1475, 1455, 1425, 1389, 1365, 1340, 1296, 1228, 1094, 1019, 966, 909, 887, 825, 736, 695, 602 cm^−1^; HRMS (FAB+): calcd. for [M + H]^+^, C_34_H_36_N_5_O_6_, 610.2666; found, 610.2668; ${\left[\alpha \right]}_{D}^{25}$ = −20.6 (*c* = 1.0, CHCl_3_).

*5′-O-(2′′,3′′,4′′,6′′-Tetra-O-benzoyl-β-d-galactopyranosyl)adenosine* (β-**24**) (Entry 1 in Table 3): A mixture of **23** (80.4 mg, 114 µmol), **13** (20.4 mg, 76.3 µmol) and **11c** (21.7 mg, 114 µmol) was co-evaporated with anhydrous pyridine (three times) and anhydrous 1,4-dioxane (three times) and dissolved in anhydrous 1,4-dioxane (760 µL). This reaction mixture was stirred under reflux conditions for 1 h and concentrated under reduced pressure. The resulting mixture was stirred with activated 4 Å molecular sieves (150 mg) in anhydrous EtCN (1.50 mL) at room temperature for 30 min and then cooled to −40 °C, to which *p*-TolSCl (30.3 µL, 229 µmol) and AgOTf (117.6 mg, 458 µmol) were added at the same temperature. After stirring for 1.5 h at −40 °C, the reaction mixture was quenched with saturated aqueous NaHCO_3_, diluted with CHCl_3_ and filtered through Celite. The organic layer was washed with saturated aqueous NaHCO_3_ and brine, dried over Na_2_SO_4_, filtered and concentrated under reduced pressure. The resulting residue was purified by silica gel column chromatography (CHCl_3_/MeOH = 1/0–30/1) to give β-**24** as a colorless solid (27.4 mg, 42% yield): ^1^H NMR (400 MHz, CDCl_3_, TMS): δ = 8.46 (s, 1H), 8.07 (dd, *J* = 7.6, 2.0 Hz, 2H), 8.02–7.98 (m, 3H), 7.97–7.93 (m, 2H), 7.83–7.79 (m, 2H), 7.57–7.51 (m, 1H), 7.49 (t, *J* = 7.6 Hz, 1H), 7.45–7.32 (m, 8H), 7.20 (t, *J* = 8.0 Hz, 2H), 6.47 (brs, 2H), 6.13 (d, *J* = 6.4 Hz, 1H), 6.04 (d, *J* = 3.2 Hz, 1H), 5.90 (dd, *J* = 10.4, 8.0 Hz, 1H), 5.73 (dd, *J* = 10.4, 3.2 Hz, 1H), 4.92 (d, *J* = 8.0 Hz, 1H), 4.70 (dd, *J* = 11.2, 6.4 Hz, 1H), 4.63 (t, *J* = 5.6 Hz, 1H), 4.47–4.33 (m, 4H), 4.20 (d, *J* = 4.8 Hz, 1H), 3.77 (d, *J* = 8.4 Hz, 1H) ppm; ^13^C NMR (100 MHz, CDCl_3_, TMS): δ = 166.1, 166.1, 165.6, 165.5, 155.3, 152.0, 148.8, 139.0, 133.8, 133.5, 133.4, 133.4, 130.0, 129.8, 129.8, 129.7, 129.3, 128.7, 128.7, 128.6, 128.5, 128.4, 119.0, 101.5, 88.3, 83.9, 76.3, 72.5, 71.6, 71.2, 70.1, 70.0, 68.0, 61.8 ppm; IR (ATR): *ν* = 3345, 3203, 3070, 2929, 1721, 1639, 1602, 1585, 1475, 1452, 1421, 1316, 1259, 1177, 1092, 1066, 1025, 1002, 938, 906, 857, 799, 753, 705, 685, 649, 617 cm^−1^; HRMS (FAB+): calcd. for [M + H]^+^, C_44_H_40_N_5_O_13_, 846.2623; found, 846.2626; Anal. Calcd. for C_44_H_39_N_5_O_13_·1.5H_2_O: C, 60.55; H, 4.85; N, 8.02; found: C, 60.47; H, 4.61; N, 7.98; ${\left[\alpha \right]}_{D}^{25}$ = +13.9 (*c* = 1.0, CHCl_3_).

*6-N-Benzoyl-5′-O-(2′′,3′′,4′′,6′′-tetra-O-benzoyl-β-d-galactopyranosyl)adenosine* (β-**25**) (Entry 2 in Table 3): *O*-Glycosylation using **23** (80.5 mg, 115 µmol), **16** (28.4 mg, 76.5 µmol), **11c** (21.8 mg, 115 µmol), anhydrous 1,4-dioxane (760 µL), *p*-TolSCl (30.3 µL, 229 µmol), AgOTf (117.8 mg, 458 µmol), 4 Å molecular sieves (150 mg) and anhydrous EtCN (1.50 mL) was conducted according to the procedure used for the synthesis of β-**24**. The residue was purified by silica gel column chromatography (CHCl_3_/MeOH = 1/0–50/1) to give β-**25** as a colorless solid (21.9 mg, 30% yield for 2 steps): ^1^H NMR (300 MHz, CDCl_3_, TMS): δ = 9.21 (brs, 1H), 8.66 (s, 1H), 8.57 (s, 1H), 8.17-8.08 (m, 2H), 7.90–7.81 (m, 4H), 7.90–7.81 (m, 2H), 7.76 (d, *J* = 7.5 Hz, 2H), 7.62–7.36 (m, 11H), 7.30 (t, *J* = 7.8 Hz, 2H), 7.22 (t, *J* = 7.8 Hz, 2H), 6.15 (d, *J* = 5.1 Hz, 1H), 6.01 (d, *J* = 3.0 Hz, 1H), 5.78 (dd, *J* = 10.2, 7.5 Hz, 1H), 5.65 (dd, *J* = 10.5, 3.3 Hz, 1H), 5.49 (brs, 1H), 4.88 (d, *J* = 7.8 Hz, 1H), 4.76–4.59 (m, 2H), 4.43 (dd, *J* = 11.1, 6.3 Hz, 1H), 4.38–4.21 (m, 4H), 3.81 (dd, *J* = 10.5, 2.7 Hz, 1H), 3.51 (s, 1H) ppm; ^13^C NMR (75 MHz, CDCl_3_, TMS): δ = 166.1, 165.5, 165.5, 164.6, 152.2, 151.0, 149.4, 141.7, 133.7, 133.5, 133.4, 132.8, 130.2, 129.8, 129.6, 129.3, 128.9, 128.8, 128.8, 128.7, 128.6, 128.5, 128.3, 127.9, 122.8, 101.5, 89.4, 84.2, 75.8, 71.8, 71.6, 71.2, 69.9, 69.3, 68.0, 61.9 ppm; IR (ATR): *ν* = 3336, 3066, 2938, 1721, 1612, 1603, 1584, 1510, 1489, 1452, 1406, 1316, 1250, 1177, 1092, 1066, 1025, 1002, 938, 901, 858, 824, 798, 755, 704, 685, 644, 616 cm^−1^; HRMS (FAB+): calcd. for [M + H]^+^, C_51_H_44_N_5_O_14_, 950.2885; found, 950.2885; Anal. Calcd. for C_51_H_43_N_5_O_14_·1.5H_2_O: C, 62.70; H, 4.75; N, 7.17; found: C, 62.80; H, 4.57; N, 7.22; ${\left[\alpha \right]}_{D}^{25}$ = +4.88 (*c* = 1.0, CHCl_3_).

*5′-O-(2′′,3′′,4′′,6′′-Tetra-O-benzoyl-β-d-galactopyranosyl)guanosine* (β-**26**) (Entry 3 in Table 3): *O*-Glycosylation using **23** (80.5 mg, 115 µmol), **17** (21.6 mg, 76.3 µmol), **11c** (21.8 mg, 115 µmol), anhydrous 1,4-dioxane (760 µL), *p*-TolSCl (30.3 µL, 229 µmol), AgOTf (117.6 mg, 458 µmol), 4 Å molecular sieves (150 mg) and anhydrous EtCN (1.50 mL) was conducted according to the procedure used for the synthesis of β-**24**. The residue was purified by silica gel column chromatography (CHCl_3_/MeOH = 1/0–8/1) to give β-**26** as a colorless solid (8.1 mg, 12% yield for 2 steps): ^1^H NMR (400 MHz, DMSO-*d*_6_, TMS): δ = 10.68 (s, 1H), 8.11-8.07 (m, 2H), 8.05 (s, 1H), 7.94 (d, *J* = 8.4 Hz, 2H), 7.85 (d, *J* = 8.4 Hz, 2H), 7.76–7.69 (m, 3H), 7.69–7.63 (m, 3H), 7.62–7.48 (m, 4H), 7.43 (t, *J* = 7.6 Hz, 2H), 7.35 (t, *J* = 7.6 Hz, 2H), 6.51 (s, 2H), 5.91 (d, *J* = 3.2 Hz, 1H), 5.86 (dd, *J* = 10.4, 3.2 Hz, 1H), 5.70 (d, *J* = 6.0 Hz, 1H), 5.60 (t, *J* = 10.0 Hz, 1H), 5.39 (d, *J* = 6.4 Hz, 1H), 5.22 (d, *J* = 7.6 Hz, 1H), 5.19 (d, *J* = 3.6 Hz, 1H), 4.69 (t, *J* = 6.4 Hz, 1H), 4.52 (dd, *J* = 11.2, 2.8 Hz, 1H), 4.42 (dd, *J* = 11.2, 6.8 Hz, 1H), 4.37 (dd, *J* = 11.2, 6.0 Hz, 1H), 4.10 (d, *J* = 8.8, 1H), 4.03 (d, *J* = 2.8 Hz, 1H), 3.92 (d, *J* = 2.8 Hz, 1H), 3.82 (dd, *J* = 10.8, 4.0 Hz, 1H) ppm; ^13^C NMR (100 MHz, DMSO-*d*_6_, TMS): δ = 165.1, 165.1, 165.1, 164.4, 156.8, 153.6, 151.4, 135.1, 133.8, 133.7, 133.5, 133.5, 129.4, 129.2, 129.1, 129.0, 129.0, 128.8, 128.7, 128.7, 128.7, 128.6, 128.6, 128.4, 116.6, 99.9, 86.4, 83.1, 73.8, 71.2, 70.7, 70.0, 69.6, 68.4, 61.7 ppm; IR (ATR): *ν* = 3332, 3128, 3065, 2935, 1724, 1673, 1638, 1602, 1584, 1572, 1538, 1491, 1452, 1350, 1316, 1261, 1177, 1092, 1067, 1025, 1002, 938, 904, 857, 801, 781, 755, 706, 686, 638, 617 cm^−1^; HRMS (FAB+): calcd. for [M + H]^+^, C_44_H_40_N_5_O_14_, 862.2572; found, 862.2573; Anal. Calcd. for C_44_H_39_N_5_O_14_·1.5H_2_O: C, 59.46; H, 4.76; N, 7.88; found: C, 59.52; H, 4.62; N, 7.87; ${\left[\alpha \right]}_{D}^{24}$ = +11.3 (*c* = 1.0, DMSO).

*2-N-Isobutyryl-5′-O-(2′′,3′′,4′′,6′′-tetra-O-benzoyl-β-d-galactopyranosyl) guanosine* (β-**27**) (Entry 4 in Table 3): Glycosylation using **23** (80.5 mg, 115 µmol), **18** (27.0 mg, 76.4 µmol), **11c** (21.8 mg, 115 µmol), anhydrous 1,4-dioxane (760 µL), *p*-TolSCl (30.3 µL, 229 µmol), AgOTf (117.8 mg, 458 µmol), 4 Å molecular sieves (150 mg) and anhydrous EtCN (1.50 mL) was conducted according to the procedure used for the synthesis of β-**24**. The residue was purified by silica gel column chromatography (CHCl_3_/MeOH = 1/0–20/1) to give β-**27** as a colorless solid (31.4 mg, 44% yield for 2 steps): ^1^H NMR (300 MHz, CDCl_3_, TMS): δ = 12.11 (s, 1H), 10.33 (s, 1H), 8.09–7.90 (m, 6H), 7.88 (s, 1H), 7.79–7.67 (m, 2H), 7.58–7.31 (m, 10H), 7.24 (t, *J* = 7.8 Hz, 2H), 6.20 (brs, 1H), 6.00 (d, *J* = 3.3 Hz, 1H), 5.79 (dd, *J* = 10.5, 7.5 Hz, 1H), 5.72–5.60 (m, 2H), 4.98 (d, *J* = 5.1 Hz, 1H), 4.84 (d, *J* = 8.1 Hz, 1H), 4.67 (dd, *J* = 10.5, 5.1 Hz, 1H), 4.42–4.24 (m, 3H), 4.16 (d, *J* = 2.7 Hz, 1H), 4.05 (brs, 2H), 3.72 (d, *J* = 8.1 Hz, 1H), 2.62–2.49 (m, 1H), 1.13 (d, *J* = 6.9 Hz, 3H), 0.94 (d, *J* = 6.6 Hz, 3H) ppm; ^13^C NMR (75 MHz, CDCl_3_, TMS): δ = 179.6, 166.5, 166.0, 165.5, 165.4, 155.6, 148.7, 147.8, 139.4, 133.8, 133.4, 129.9, 129.7, 129.6, 129.2, 128.8, 128.6, 128.5, 128.3, 120.6, 101.8, 89.4, 83.7, 72.5, 71.4, 71.2, 70.1, 69.4, 67.9, 61.6, 36.1, 18.8, 18.5 ppm; IR (ATR): *ν* = 3201, 3067, 2974, 2936, 1720, 1677, 1602, 1560, 1475, 1452, 1404, 1376, 1350, 1316, 1258, 1178, 1156, 1092, 1066, 1026, 1002, 949, 908, 856, 802, 784, 752, 706, 687, 642, 617 cm^−1^; HRMS (FAB+): calcd. for [M + H]^+^, C_48_H_46_N_5_O_15_, 932.2990; found, 932.2990; Anal. Calcd. for C_48_H_45_N_5_O_15_·1.5H_2_O: C, 60.12; H, 5.05; N, 7.30; found: C, 60.29; H, 4.86; N, 7.34; ${\left[\alpha \right]}_{D}^{25}$ = +25.9 (*c* = 1.0, CHCl_3_).

*5′-O-(2′′,3′′,4′′,6′′-Tetra-O-benzoyl-β-d-galactopyranosyl)uridine* (β-**28**) (Entry 5 in Table 3): *O*-Glycosylation using **23** (80.4 mg, 114 µmol), **10** (18.6 mg, 76.2 µmol), **11c** (21.7 mg, 114 µmol), anhydrous 1,4-dioxane (760 µL), *p*-TolSCl (30.3 µL, 229 µmol), AgOTf (117.6 mg, 458 µmol), 4 Å molecular sieves (150 mg) and anhydrous EtCN (1.50 mL) was conducted according to the procedure used for the synthesis of β-**24**. The residue was purified by silica gel column chromatography (CHCl_3_/MeOH = 1/0–40/1) to give β-**28** as a colorless solid (26.1 mg, 42% yield for 2 steps): ^1^H NMR (400 MHz, CDCl_3_, TMS): δ = 9.91 (s, 1H), 8.10–8.05 (m, 2H), 8.04–7.99 (m, 2H), 7.95–7.86 (m, 3H), 7.79–7.75 (m, 2H), 7.63 (t, *J* = 7.6 Hz, 1H), 7.59–7.49 (m, 3H), 7.50–7.38 (m, 4H), 7.33 (t, *J* = 7.6 Hz, 2H), 7.23 (t, *J* = 7.6 Hz, 2H), 6.02 (d, *J* = 2.8 Hz, 1H), 5.92–5.84 (m, 2H), 5.77 (dd, *J* = 10.4, 8.0 Hz, 1H), 5.67 (dd, *J* = 10.8, 3.6 Hz, 1H), 5.03 (d, *J* = 4.0 Hz, 1H), 4.91 (d, *J* = 7.6 Hz, 1H), 4.71 (dd, *J* = 10.8, 6.0 Hz, 1H), 4.49–4.39 (m, 3H), 4.23 (d, *J* = 4.4 Hz, 1H), 4.13–4.02 (m, 2H), 3.79 (d, *J* = 10.0 Hz, 1H), 3.39 (d, *J* = 5.6 Hz, 1H) ppm; ^13^C NMR (100 MHz, CDCl_3_, TMS): δ = 166.1, 165.5, 165.5, 165.5, 163.6, 151.3, 140.2, 133.9, 133.6, 133.4, 129.8, 129.8, 129.7, 129.3, 128.9, 128.7, 128.6, 128.6, 128.5, 128.4, 102.6, 101.5, 90.6, 83.4, 75.2, 71.7, 71.2, 70.0, 69.8, 68.2, 68.1, 61.9 ppm; IR (ATR): *ν* = 3356, 3069, 2972, 1720, 1687, 1602, 1585, 1492, 1452, 1383, 1316, 1261, 1178, 1093, 1067, 1027, 1002, 907, 858, 806, 763, 706, 686, 617 cm^−1^; HRMS (FAB+): calcd. for [M + H]^+^, C_43_H_39_N_2_O_15_, 823.2350; found, 823.2352; Anal. Calcd. for C_43_H_38_N_2_O_15_·H_2_O: C, 61.43; H, 4.80; N, 3.33; found: C, 61.45; H, 4.70; N, 3.38; ${\left[\alpha \right]}_{D}^{25}$ = +50.7 (*c* = 1.0, CHCl_3_).

*5-Metyl-5′-O-(2′′,3′′,4′′,6′′-tetra-O-benzoyl-β-d-galactopyranosyl)uridine* (β-**29**) (Entry 6 in Table 3): *O*-Glycosylation using **23** (80.5 mg, 115 µmol), **19** (19.7 mg, 76.3 µmol), **11c** (21.8 mg, 115 µmol), anhydrous 1,4-dioxane (760 µL), *p*-TolSCl (30.3 µL, 229 µmol), AgOTf (117.6 mg, 458 µmol), 4 Å molecular sieves (150 mg) and anhydrous EtCN (1.50 mL) was conducted according to the procedure used for the synthesis of β-**24**. The residue was purified by silica gel column chromatography (CHCl_3_/MeOH = 1/0–40/1) to give β-**29** as a colorless solid (33.8 mg, 53% yield for 2 steps): ^1^H NMR (400 MHz, CDCl_3_, TMS): δ = 10.04 (s, 1H), 8.09–8.05 (m, 2H), 8.04–8.00 (m, 2H), 7.97–7.93 (m, 2H), 7.79–7.74 (m, 2H), 7.67 (s, 1H), 7.61–7.52 (m, 2H), 7.50–7.37 (m, 6H), 7.34 (t, *J* = 7.6 Hz, 2H), 7.20 (t, *J* = 7.6 Hz, 2H), 6.03 (d, *J* = 3.6 Hz, 1H), 5.88 (d, *J* = 4.4 Hz, 1H), 5.81 (dd, *J* = 10.4, 7.6 Hz, 1H), 5.71 (dd, *J* = 10.4, 3.2 Hz, 1H), 5.08 (s, 1H), 4.90 (d, *J* = 7.6 Hz, 1H), 4.70 (dd, *J* = 11.2, 6.4 Hz, 1H), 4.50–4.37 (m, 3H), 4.21 (d, *J* = 4.4 Hz, 1H), 4.09 (dd, *J* = 10.0, 4.4 Hz, 1H), 4.01 (dd, *J* = 10.0, 4.8 Hz, 1H), 3.77 (d, *J* = 9.2 Hz, 1H), 3.41 (d, *J* = 4.8 Hz, 1H), 2.06 (s, 3H) ppm; ^13^C NMR (100 MHz, CDCl_3_, TMS): δ = 166.1, 165.6, 165.6, 165.5, 164.2, 151.3, 136.1, 133.8, 133.6, 133.4, 129.9, 129.8, 129.7, 129.7, 129.3, 128.9, 128.7, 128.6, 128.5, 128.5, 128.3, 111.3, 102.2, 89.8, 83.3, 74.6, 71.8, 71.2, 70.2, 69.8, 69.3, 68.1, 61.9, 12.8 ppm; IR (ATR): *ν* = 3385, 3067, 2930, 1720, 1686, 1602, 1585, 1492, 1468, 1452, 1386, 1349, 1316, 1259, 1177, 1092, 1066, 1025, 1002, 937, 909, 858, 802, 793, 755, 705, 685, 616 cm^−1^; HRMS (FAB+): calcd. for [M + H]^+^, C_44_H_41_N_2_O_15_, 837.2507; found, 837.2510; Anal. Calcd. for C_44_H_40_N_2_O_15_·H_2_O: C, 61.82; H, 4.95; N, 3.28; found: C, 61.70; H, 4.85; N, 3.30; ${\left[\alpha \right]}_{D}^{25}$ = +28.1 (*c* = 1.0, CHCl_3_).

*5-Fluoro-5′-O-(2′′,3′′,4′′,6′′-tetra-O-benzoyl-β-d-galactopyranosyl)uridine* (β-**30**) (Entry 7 in Table 3): *O*-Glycosylation using **23** (80.4 mg, 114 µmol), **20** (20.0 mg, 76.3 µmol), **11c** (21.7 mg, 114 µmol), anhydrous 1,4-dioxane (760 µL), *p*-TolSCl (30.3 µL, 229 µmol), AgOTf (117.6 mg, 458 µmol), 4 Å molecular sieves (150 mg) and anhydrous EtCN (1.50 mL) was conducted according to the procedure used for the synthesis of β-**24**. The residue was purified by silica gel column chromatography (CHCl_3_ then AcOEt/CHCl_3_ = 1/1) to give β-**30** as a colorless solid (38.8 mg, 61% yield for 2 steps): ^1^H NMR (300 MHz, CDCl_3_, TMS): δ = 9.81 (brs, 1H), 8.10 (t, *J* = 7.2 Hz, 3H), 8.04–7.99 (m, 2H), 7.94–7.89 (m, 2H), 7.78–7.73 (m, 2H), 7.64–7.48 (m, 4H), 7.47–7.37 (m, 4H), 7.33 (t, *J* = 7.5 Hz, 2H), 7.21 (t, *J* = 7.2 Hz, 2H), 6.03 (d, *J* = 3.0 Hz, 1H), 5.88 (d, *J* = 3.9 Hz, 1H), 5.78 (dd, *J* = 10.2, 7.5 Hz, 1H), 5.71 (dd, *J* = 10.2, 3.3 Hz, 1H), 4.86 (d, *J* = 7.2 Hz, 1H), 4.72 (dd, *J* = 11.1, 6.3 Hz, 1H), 4.51 (brs, 1H), 4.52–4.33 (m, 3H), 4.27 (d, *J* = 3.3 Hz, 1H), 4.19 (t, *J* = 4.8 Hz, 1H), 4.02 (s, 1H), 3.74 (d, *J* = 9.6 Hz, 1H), 3.32 (brs, 1H) ppm; ^13^C NMR (100 MHz, CDCl_3_, TMS): δ = 166.1, 165.8, 165.7, 165.5, 157.0 (d, ^2^*J*_CF_ = 26.4 Hz), 149.9, 140.8 (d, ^1^*J*_CF_ = 237.0 Hz), 133.9, 133.7, 133.4, 130.0, 129.8, 129.7, 129.3, 128.8, 128.7, 128.6, 128.5, 128.4, 124.9 (d, ^2^*J*_CF_ = 35.5 Hz), 101.7, 90.5, 84.0, 75.3, 71.8, 71.0, 70.9, 69.9, 69.0, 67.9, 61.8 ppm; ^19^F NMR (376 MHz, CDCl_3_, TFA): δ = −164.57 (s) ppm; IR (ATR): *ν* = 3447, 3074, 2941, 1715, 1602, 1585, 1493,1452, 1351, 1317, 1258, 1178, 1092, 1066, 1026, 1002, 936, 894, 858, 800, 753, 706, 687, 617 cm^−1^; HRMS (FAB+): calcd. for [M + Na]^+^, C_43_H_37_FN_2_O_15_Na, 863.2076; found, 863.2072; Anal. Calcd. for C_43_H_37_FN_2_O_15_·H_2_O: C, 60.14; H, 4.58; N, 3.26; found: C, 60.02; H, 4.41; N, 3.32; ${\left[\alpha \right]}_{D}^{25}$ = +37.2 (*c* = 1.0, CHCl_3_).

*5′-O-(2′′,3′′,4′′,6′′-Tetra-O-benzoyl-β-d-galactopyranosyl)cytidine* (β-**31**) (Entry 8 in Table 3): *O*-Glycosylation using **23** (80.4 mg, 114 µmol), **21** (18.5 mg, 76.1 µmol), **11c** (21.7 mg, 114 µmol), anhydrous 1,4-dioxane (760 µL), *p*-TolSCl (30.3 µL, 229 µmol), AgOTf (117.6 mg, 458 µmol), 4 Å molecular sieves (150 mg) and anhydrous EtCN (1.50 mL) was conducted according to the procedure used for the synthesis of β-**24**. The residue was purified by silica gel column chromatography (CHCl_3_/MeOH = 1/0–10/1) to give β-**31** as a colorless solid (34.1 mg, 55% yield for 2 steps): ^1^H NMR (300 MHz, acetone-*d*_6_, TMS): δ = 8.13-8.08 (m, 2H), 8.06–8.01 (m, 2H), 7.97–7.92 (m, 2H), 7.88 (d, *J* = 7.5 Hz, 1H), 7.76 (dd, *J* = 8.4, 1.1 Hz, 2H), 7.73-7.65 (m, 1H), 7.65-7.56 (m, 3H), 7.56-7.44 (m, 4H), 7.39 (t, *J* = 7.2 Hz, 2H), 7.32 (t, *J* = 7.5 Hz, 2H), 6.90 (brs, 2H), 6.10 (dd, *J* = 3.0, 0.9 Hz, 1H), 5.97 (d, *J* = 7.8 Hz, 1H), 5.90 (d, *J* = 7.8 Hz, 1H), 5.87–5.77 (m, 2H), 5.33 (d, *J* = 7.8 Hz, 1H), 4.78 (t, *J* = 6.3 Hz, 1H), 4.70 (dd, *J* = 10.8, 6.0 Hz, 1H), 4.54 (dd, *J* = 10.8, 6.6 Hz, 1H), 4.38 (dd, *J* = 11.1, 1.8 Hz, 1H), 4.19–4.12 (m, 1H), 4.06 (t, *J* = 4.5 Hz, 1H), 4.02–3.93 (m, 2H) ppm; ^13^C NMR (100 MHz, acetone-*d*_6_, TMS): δ = 166.9, 166.4, 166.3, 166.2, 165.8, 157.0, 142.1, 134.6, 134.3, 134.3, 134.2, 130.7, 130.5, 130.4, 130.3, 130.2, 130.2, 130.1, 129.8, 129.5, 129.4, 129.3, 102.2, 95.4, 91.6, 84.0, 76.4, 72.8, 72.0, 71.1, 70.9, 69.8, 69.7, 62.8 ppm; IR (ATR): *ν* = 3350, 3208, 3072, 2935, 1723, 1642, 1602, 1529, 1486, 1452, 1349, 1316, 1259, 1178, 1092, 1065, 1025, 1002, 940, 909, 857, 788, 753, 705, 685, 616 cm^−1^; HRMS (FAB+): calcd. for [M + H]^+^, C_43_H_40_N_3_O_14_, 822.2510; found, 822.2507; Anal. Calcd. for C_43_H_39_N_3_O_14_·1.5H_2_O: C, 60.85; H, 4.99; N, 4.95; found: C, 60.87; H, 4.72; N, 4.97; ${\left[\alpha \right]}_{D}^{25}$ = +62.4 (*c* = 1.0, CHCl_3_).

*4-N-Benzoyl-5′-O-(2′′,3′′,4′′,6′′-tetra-O-benzoyl-β-d-galactopyranosyl)cytidine* (β-**32**) (Entry 9 in Table 3): *O*-glycosylation using **23** (80.6 mg, 115 µmol), **22** (26.6 mg, 76.6 µmol), **11c** (21.8 mg, 115 µmol), anhydrous 1,4-dioxane (760 µL), *p*-TolSCl (30.3 µL, 229 µmol), AgOTf (117.8 mg, 458 µmol), 4 Å molecular sieves (150 mg) and anhydrous EtCN (1.50 mL) was conducted according to the procedure used for the synthesis of β-**24**. The residue was purified by silica gel column chromatography (CHCl_3_/MeOH = 1/0–50/1) to give β-**32** as a colorless solid (28.0 mg, 40% yield for 2 steps): ^1^H NMR (400 MHz, CDCl_3_, TMS): δ = 8.93 (brs, 1H), 8.28 (d, *J* = 7.6 Hz, 1H), 8.06–8.01 (m, 4H), 7.91 (dd, *J* = 8.4, 1.6 Hz, 2H), 7.87 (d, *J* = 7.2 Hz, 2H), 7.73 (dd, *J* = 8.0, 1.6 Hz, 2H), 7.68 (brs, 1H), 7.60–7.53 (m, 2H), 7.53–7.37 (m, 9H), 7.32 (t, *J* = 8.0 Hz, 2H), 7.19 (t, *J* = 8.0 Hz, 2H), 6.03 (d, *J* = 3.2 Hz, 1H), 5.86 (d, *J* = 3.6 Hz, 1H), 5.76 (dd, *J* = 10.4, 8.0 Hz, 1H), 5.69 (dd, *J* = 10.4, 3.6 Hz, 1H), 5.54 (brs, 1H), 4.92 (d, *J* = 7.6 Hz, 1H), 4.78 (dd, *J* = 11.6, 6.4 Hz, 1H), 4.48 (dd, *J* = 11.2, 6.4 Hz, 1H), 4.43–4.35 (m, 3H), 4.14 (t, *J* = 4.4 Hz, 1H), 4.10 (d, *J* = 3.6 Hz, 1H), 3.81 (dd, *J* = 11.6, 2.4 Hz, 1H), 3.66 (brs, 1H) ppm; ^13^C NMR (100 MHz, CDCl_3_, TMS): δ = 166.1, 165.6, 165.5, 165.3, 162.6, 144.7, 133.6, 133.4, 133.4, 133.1, 132.9, 129.9, 129.8, 129.8, 129.7, 129.4, 128.9, 128.8, 128.6, 128.5, 128.4, 128.3, 127.7, 101.7, 97.1, 93.1, 84.8, 76.4, 71.8, 71.3, 71.2, 69.6, 68.7, 68.1, 61.9 ppm; IR (ATR): *ν* = 3320, 3066, 2930, 1724, 1645, 1603, 1556, 1481, 1452, 1379, 1315, 1248, 1177, 1092, 1066, 1025, 1002, 938, 899, 859, 802, 787, 756, 704, 685, 616 cm^−1^; HRMS (FAB+): calcd. for [M + H]^+^, C_50_H_44_N_3_O_15_, 926.2772; found, 926.2773; Anal. Calcd. for C_50_H_43_N_3_O_15_·H_2_O: C, 63.62; H, 4.81; N, 4.45; found: C, 63.34; H, 4.71; N, 4.56; ${\left[\alpha \right]}_{D}^{25}$ = +46.6 (*c* = 1.0, CHCl_3_).

*5-Fluoro-5′-O-(2′′,3′′,4′′,6′′-tetra-O-benzoyl-β-d-glucopyranosyl)uridine* (β-**35**) (Entry 1 in Table 4): *O*-Glycosylation using **33** (80.4 mg, 114 µmol), **20** (20.0 mg, 76.2 µmol), **11c** (21.7 mg, 114 µmol), anhydrous 1,4-dioxane (760 µL), *p*-TolSCl (30.3 µL, 229 µmol), AgOTf (117.6 mg, 458 µmol), 4 Å molecular sieves (150 mg) and anhydrous EtCN (1.50 mL) was conducted according to the procedure used for the synthesis of β-**24**. The residue was purified by silica gel column chromatography (CHCl_3_/MeOH = 1/0–30/1) to give β-**35** as a colorless solid (34.5 mg, 54% yield for 2 steps): ^1^H NMR (300 MHz, CDCl_3_, TMS): δ = 9.66 (brs, 1H), 8.09 (d, *J* = 6.6 Hz, 1H), 8.04-7.98 (m, 2H), 7.91 (d, *J* = 8.1 Hz, 4H), 7.87–7.82 (m, 2H), 7.56–7.24 (m, 12H), 5.97 (t, *J* = 9.9 Hz, 1H), 5.82 (d, *J* = 3.0 Hz, 1H), 5.69 (t, *J* = 9.9 Hz, 1H), 5.48 (dd, *J* = 10.2, 7.8 Hz, 1H), 4.90 (d, *J* = 8.1 Hz, 1H), 4.70 (dd, *J* = 12.0, 3.0 Hz, 1H), 4.55 (brs, 1H), 4.52 (dd, *J* = 12.0, 4.8 Hz, 1H), 4.33 (dd, *J* = 10.8, 2.1 Hz, 1H), 4.28–4.15 (m, 3H), 4.06 (s, 1H), 3.76 (d, *J* = 9.6 Hz, 1H), 3.31 (s, 1H) ppm; ^13^C NMR (100 MHz, CDCl_3_, TMS): δ = 166.2, 165.8, 165.5, 165.0, 157.1 (d, ^2^*J*_CF_ = 26.4 Hz), 149.8, 140.7 (d, ^1^*J*_CF_ = 236.2 Hz), 133.7, 133.5, 133.4, 133.3, 129.9, 129.8, 129.7, 129.4, 128.7, 128.6, 128.5, 128.4, 128.4, 124.8 (d, ^2^*J*_CF_ = 34.6 Hz), 100.8, 90.6, 83.9, 75.4, 72.7, 72.3, 71.9, 70.6, 69.6, 68.0, 62.8 ppm; ^19^F NMR (376 MHz, CDCl_3_, TFA): δ = −165.00 (s) ppm; IR (ATR): *ν* = 3426, 3072, 2953, 1716, 1602, 1585, 1493,1452, 1369, 1317, 1260, 1178, 1091, 1068, 1027, 1003, 936, 895, 855, 800, 758, 708, 687, 618 cm^−1^; HRMS (FAB+): calcd. for [M + H]^+^, C_43_H_38_FN_2_O_15_, 841.2256; found, 841.2261; Anal. Calcd. for C_43_H_37_FN_2_O_15_·1.5H_2_O: C, 59.52; H, 4.65; N, 3.23; found: C, 59.51; H, 4.47; N, 3.26; ${\left[\alpha \right]}_{D}^{25}$ = +8.39 (*c* = 1.0, CHCl_3_).

*O*-Glycosylation using glycosyl donors containing boronic acid on leaving group (Entries 1–4 in Table 5): A mixture of **41** or **42** (30.2 mg, 48.0 µmol) and **10** (7.8 mg, 31.9 µmol) or **13** (8.6 mg, 32.2 µmol) was co-evaporated with anhydrous pyridine (three times) and anhydrous 1,4-dioxane (three times) and dissolved in anhydrous 1,4-dioxane (320 µL). This reaction mixture was stirred under reflux conditions for 1 h and concentrated under reduced pressure. *O*-glycosylation and acetylation were conducted according to the procedure used for the synthesis of **12** or **14** using *p*-TolSCl (12.7 µL, 96.1 µmol), AgOTf (49.5 mg, 193 µmol), 3 Å molecular sieves (64 mg), anhydrous MeCN (640 µL), anhydrous pyridine (200 µL), Ac_2_O (10.0 equiv. based on the crude compound) and DMAP (catalytic amount).

*5′-O-(2′′,3′′,4′′-Tri-O-benzyl-α/β-d-mannopyranosyl)uridine* (**43**) (Scheme 1a): To a solution of **12** (25.2 mg, 31.4 µmol, α/β = 1.6/1) in THF (400 µL), 1 M aqueous LiOH was added at room temperature. After stirring for 2 h, the reaction mixture was neutralized with 0.1 M aqueous HCl, extracted with CHCl_3_, washed with brine, dried over Na_2_SO_4_, filtered and concentrated under reduced pressure. The residue was purified by silica gel column chromatography (CHCl_3_/MeOH = 20/1) to give α-**43** as a colorless solid (12.1 mg, 57% yield) and β-**43** as a colorless solid (7.5 mg, 35% yield): α-**43**; ^1^H NMR (300 MHz, CDCl_3_, TMS): δ = 10.11 (s, 1H), 7.39 (d, *J* = 8.4 Hz, 1H), 7.36–7.17 (m, 15H), 5.74 (d, *J* = 2.1 Hz, 1H), 5.43 (d, *J* = 8.4 Hz, 1H), 4.91 (s, 1H), 4.87 (t, *J* = 6.3 Hz, 2H), 4.75 (d, *J* = 12.0 Hz, 1H), 4.67 (d, *J* = 9.0 Hz, 1H), 4.64–4.51 (m, 3H), 4.20 (s, 1H), 4.13 (s, 1H), 4.02 (s, 2H), 3.90–3.74 (m, 4H), 3.73–3.56 (m, 4H), 3.00 (s, 1H) ppm; ^13^C NMR (100 MHz, CDCl_3_, TMS): δ = 163.7, 151.1, 139.7, 138.0, 137.8, 128.5, 128.4, 128.4, 128.3, 127.9, 127.8, 127.7, 127.6, 102.3, 98.3 (C_1”_, ^1^*J*_CH_ = 169.4 Hz), 90.1, 82.6, 79.7, 75.1, 75.0, 74.8, 74.7, 73.1, 73.0, 72.4, 69.9, 66.6, 62.3 ppm; IR (ATR): *ν* = 3384, 3064, 3032, 2924, 2879, 1683, 1497, 1455, 1389, 1364, 1321, 1269, 1210, 1068, 1027, 909, 864, 845, 810, 735, 697 cm^−1^; HRMS (FAB+): calcd. for [M + H]^+^, C_36_H_41_N_2_O_11_, 677.2710; found, 677.2709; Anal. Calcd. for C_36_H_40_N_2_O_11_·H_2_O: C, 62.24; H, 6.09; N, 4.03; found: C, 62.36; H, 6.01; N, 4.13; ${\left[\alpha \right]}_{D}^{25}$ = +42.7 (*c* = 0.2, CHCl_3_); β-**43**; ^1^H NMR (300 MHz, CD_3_OD, TMS): δ = 7.96 (d, *J* = 8.1 Hz, 1H), 7.41–7.20 (m, 15H), 5.96 (d, *J* = 5.4 Hz, 1H), 5.34 (d, *J* = 8.1 Hz, 1H), 4.92–4.84 (m, 2H), 4.75–4.55 (m, 5H), 4.28 (t, *J* = 5.7 Hz, 1H), 4.22 (dd, *J* = 5.1, 3.3 Hz, 1H), 4.18–4.10 (m, 2H), 4.08 (d, *J* = 2.1 Hz, 1H), 3.86–3.73 (m, 3H), 3.67 (dd, *J* = 9.6, 2.7 Hz, 1H), 3.60 (dd, *J* = 11.7, 6.3 Hz, 1H), 3.35–3.26 (m, 1H) ppm; ^13^C NMR (100 MHz, CD_3_OD, TMS): δ = 166.1, 152.6, 143.4, 139.8, 139.7, 129.7, 129.4, 129.3, 129.3, 128.9, 128.8, 128.7, 103.0, 101.8 (C_1”_, ^1^*J*_CH_ = 156.1 Hz), 89.9, 85.2, 84.1, 77.9, 76.9, 76.2, 76.0, 75.8, 75.6, 73.0, 72.2, 70.0, 62.8 ppm; IR (ATR): *ν* = 3387, 3063, 3032, 2926, 2874, 1673, 1498, 1456, 1401, 1364, 1316, 1274, 1249, 1211, 1179, 1072, 1027, 906, 866, 811, 786, 736, 696 cm^−1^; HRMS (FAB+): calcd. for [M + H]^+^, C_36_H_41_N_2_O_11_, 677.2710; found, 677.2709; Anal. Calcd. for C_36_H_40_N_2_O_11_·H_2_O: C, 62.24; H, 6.09; N, 4.03; found: C, 62.29; H, 5.86; N, 4.20; ${\left[\alpha \right]}_{D}^{23}$ = −67.6 (*c* = 0.5, CH_3_OH).

*5′-O-α/β-d-Mannopyranosyl)uridine* (**44**) (Scheme 1a): A mixture of α-**43** (19.2 mg, 28.4 µmol), 10% Pd/C (19.0 mg) in MeOH (540 µL) was vigorously stirred for 22 h at room temperature under a H_2_ atmosphere. The mixture was filtered through Celite with MeOH and H_2_O, and then, the filtrate was concentrated under reduced pressure to give α-**44** as a colorless solid (11.4 mg, 99% yield): α-**44**; ^1^H NMR (300 MHz, D_2_O, TSP): δ = 7.89 (d, *J* = 8.1 Hz, 1H), 5.96–5.87 (m, 2H), 4.96 (s, 1H), 4.33 (s, 3H), 4.06–3.96 (m, 2H), 3.90 (t, *J* = 10.5 Hz, 2H), 3.57–3.84 (m, 4H) ppm; ^13^C NMR (75 MHz, D_2_O, 1,4-dioxane): δ = 166.8, 152.1, 142.0, 102.5, 100.3, 90.4, 82.9, 74.6, 73.6, 71.2, 70.6, 69.8, 67.2, 66.3, 61.5 ppm; IR (ATR): *ν* = 3289, 2935, 2502, 1666, 1466, 1397, 1273, 1199, 1129, 1104, 1050, 1025, 912, 868, 810, 801, 765, 720, 676, 622 cm^−1^; HRMS (FAB+): calcd. for [M + Na]^+^, C_15_H_22_N_2_O_11_Na, 429.1121; found, 429.1118; Anal. Calcd. for C_15_H_22_N_2_O_11_·2.75H_2_O: C, 39.52; H, 6.08; N, 6.14; found: C, 39.58; H, 5.93; N, 5.81; ${\left[\alpha \right]}_{D}^{24}$ = +29.3 (*c* = 0.8, H_2_O).

Cleavage of benzyl groups using β-**43** (17.8 mg, 26.3 µmol), 10% Pd/C (18.0 mg) and MeOH (500 µL) was conducted according to the procedure for synthesis of α-**44** to give the β-**44** as a colorless solid (10.5 mg, 98% yield): β-**44**; ^1^H NMR (300 MHz, D_2_O, TSP): δ = 8.05 (d, *J* = 8.1 Hz, 1H), 5.96 (d, *J* = 4.2 Hz, 1H), 5.89 (d, *J* = 8.1 Hz, 1H), 4.74 (s, 1H), 4.49–4.12 (m, 4H), 4.05 (s, 1H), 3.95 (d, *J* = 12.3 Hz, 1H), 3.88 (d, *J* = 11.7 Hz, 1H), 3.75 (dd, *J* = 11.7, 6.6 Hz, 1H), 3.70–3.52 (m, 2H), 3.40 (t, *J* = 6.6 Hz, 1H) ppm; ^13^C NMR (75 MHz, D_2_O, 1,4-dioxane): δ = 167.8, 153.0, 142.7, 103.0, 100.9, 89.7, 83.6, 76.9, 74.4, 73.5, 70.9, 70.4, 69.0, 67.5, 61.7 ppm; IR (ATR): *ν* = 3288, 2933, 2503, 1670, 1510, 1465, 1390, 1266, 1133, 1053, 1023, 879, 815, 790, 764, 714, 632, 616 cm^−1^; HRMS (FAB+): calcd. for [M + Na]^+^, C_15_H_22_N_2_O_11_Na, 429.1121; found, 429.1119; Anal. Calcd. for C_15_H_22_N_2_O_11_·2.6H_2_O: C, 39.76; H, 6.05; N, 6.18; found: C, 40.15; H, 6.00; N, 5.80; ${\left[\alpha \right]}_{D}^{25}$ = −10.6 (*c* = 0.8, H_2_O).

*5-Fluoro-5′-O-(β-d-galactopyranosyl)uridine* (β-**45**) (Scheme 1b): A mixture of β-**30** (25.2 mg, 30.0 µmol) and 10 M MeNH_2_ in MeOH was stirred at 0 °C for 2 h and then allowed to warm to room temperature. After stirring for 13 h, the reaction mixture was concentrated under reduced pressure. The residue was dissolved in H_2_O, and the *N*-methylbenzamide was removed by successive washing of the aqueous phase with CH_2_Cl_2_. The aqueous layer was concentrated under reduced pressure. The residue was purified by preparative HPLC (H_2_O (0.1%TFA)) to give β-**45** as a colorless amorphous solid (7.9 mg, 62% yield): ^1^H NMR (300 MHz, D_2_O, TSP): δ = 8.18 (d, *J* = 6.6 Hz, 1H), 5.94 (d, *J* = 1.8 Hz, 1H), 4.51 (d, *J* = 7.2 Hz, 1H), 4.48–4.24 (m, 4H), 3.98–3.86 (m, 2H), 3.86–3.77 (m, 2H), 3.77–3.69 (m, 1H), 3.69–3.58 (m, 2H) ppm; ^13^C NMR (100 MHz, D_2_O, 1,4-dioxane): δ = 160.1 (d, ^2^*J*_CF_ = 26.4 Hz), 150.8, 141.4 (d, ^1^*J*_CF_ = 232.1 Hz), 126.3 (d, ^2^*J*_CF_ = 38.1 Hz), 103.7, 89.9, 83.6, 75.9, 74.4, 73.4, 71.5, 69.9, 69.2, 68.9, 61.6 ppm; ^19^F NMR (376 MHz, D_2_O, TFA): δ = −166.73 (s) ppm; IR (ATR): *ν* = 3357, 3075, 2935, 2827, 1661, 1477, 1398, 1365, 1258, 1202, 1035, 952, 921, 890, 843, 793, 750, 722, 697 cm^−1^; HRMS (FAB+): calcd. for [M + Na]^+^, C_15_H_21_FN_2_O_11_Na, 447.1027; found, 447.1030; ${\left[\alpha \right]}_{D}^{25}$ = +17.6 (*c* = 0.3, H_2_O).

^1^H, ^11^B and ^19^F NMR measurements of mixtures of uridine (**10**) and boronic acid (**11c**) (Figure 3): A mixture of **10** (34.3 mg, 140 µmol) and **11c** (40.0 mg, 211 µmol) was co-evaporated with anhydrous pyridine (three times) and anhydrous 1,4-dioxane (three times). The resulting residue was dissolved in anhydrous 1,4-dioxane (1.40 mL) and then stirred under reflux conditions for 1 h. The reaction mixture (140 µL) was separated and concentrated under reduced pressure. The residue **46** dissolved in CD_3_CN (640 µL) was measured by a ^1^H, ^11^B and ^19^F NMR spectrometers. **11c** was treated under the same conditions as were used to prepare **48** for the ^11^B and ^19^F NMR measurements.

## 4. Conclusions

We report herein on the synthesis of disaccharide nucleosides utilizing the temporary protection of the 2′,3′-*cis*-diol of ribonucleosides by a boronic ester. The glycosylation of the uridine **10**, which is temporarily protected by a boronic acid, with the thioglycoside **9** using a *p*-TolSCl/AgOTf promoter system followed by acetylation gave the disaccharide nucleoside **12** containing a 1′′,5′-glycosidic linkage in reasonable chemical yield. This synthetic method was applied to the glycosylation of protected or unprotected adenosine, guanosine, uridine or cytidine, **10**, **13**, **16**–**22**, with the galactosyl donor **23** to afford the desired products in moderate chemical yields. *O*-glycosylations of 5-fluorouridine **20** with the glucosyl donor **33**, the galactosyl donor **23** and the mannosyl donor **34** were also conducted. The introduction of a boronic acid on the phenylthio leaving group had only a negligible effect on the reactivity and stereoselectivity of the system. The deprotection of compounds **12** and β-**30** was also demonstrated to give the corresponding deprotected compounds α-**44** and β-**44** from **12** and β-**45** from β-**30**. Because 5-fluorouridine and 5-fluorouracil have been reported to have anticancer, antivirus and antibacterial activities [24,68,72,73,74,75,76,77,78], β-**45** and its analogs represent potentially new drug candidates.

Finally, ^1^H, ^11^B and ^19^F NMR measurements of a mixture of uridine **10** and 4-(trifluoromethyl)phenylboronic acid **11c** suggest that the 2’ and 3’ hydroxyl groups of **10** react with **11c** to form the cyclic boronic ester intermediate **47**, as expected, resulting in selective *O*-glycosylation of the ribonucleoside acceptors at the 5’-position.

These results afford important and useful information regarding the concise and short-step synthesis of various biologically-active disaccharide nucleoside derivatives via the *O*-glycosylation of temporarily-protected nucleosides and related compounds.

## Figures and Tables

**Table 1:**
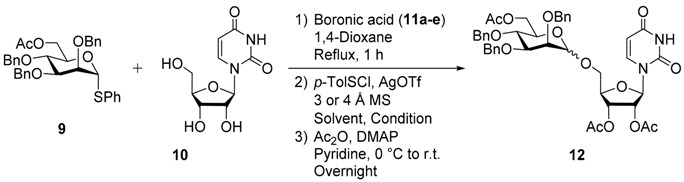
*O*-glycosylation of uridine **10** with the thiomannoside **9** in the absence and presence of boronic acid.

Entry	Boronic Acid *^b^*	Solvent	Condition	Yield (for 3 Steps) *^c^*
1 ***^a^***	-	MeCN	−20 °C, 1.5 h	<16% (complex mixture)
2 *^**a,**d^*	PhB(OH)_2_ (**11a**)	MeCN	−20 °C, 1.5 h	41% (α/β = 1.6/1)
3 *^**a,**e^*	**11a**	MeCN	−20 °C, 1.5 h	45% (α/β = 1.6/1)
4 *^**a,**e^*	4-MeOPhB(OH)_2_ (**11b**)	MeCN	−20 °C, 1.5 h	39% (α/β = 1.8/1)
5 *^**a,**e^*	4-CF_3_PhB(OH)_2_ (**11c**)	MeCN	−20 °C, 1.5 h	51% (α/β = 1.8/1)
6 *^**a,**e^*	2,4-F_2_PhB(OH)_2_ (**11d**)	MeCN	−20 °C, 1.5 h	46% (α/β = 1.8/1)
7 *^**a,**e^*	**11c**	1,4-Dioxane	r.t., 1.5 h	27% (α/β = 3.3/1)
8 *^**a,**e^*	**11c**	CH_2_Cl_2_	−40 °C, 1.5 h	trace
9 *^**a,**e^*	**11c**	EtCN	−40 °C, 1.5 h	61% (α/β = 1.6/1)
10 ***^e,f^***	**11c**	EtCN	−40 °C, 1.5 h	57% (α/β = 1.5/1)
11 *^**a,**e^*	4-CH_3_(CH_2_)_5_PhB(OH)_2_ (**11e**)	EtCN	−40 °C, 1.5 h	30% (α/β = 1.6/1)

*^a^* Glycosylation reactions were carried out in the presence of 1.5 equivalents of **9**, 3.0 equivalents of *p*-TolSCl and 6.0 equivalents of AgOTf against **10**. Acetylation reactions were carried out in the presence of *ca.* 10 equivalents of Ac_2_O (acetic anhydride) and catalytic amount of DMAP. *^b^* Stoichiometry of **11** was 1.5 equivalents against **10**. *^c^* The α/β ratio was determined by ^1^H NMR. *^d^* A mixture of **10** and **11a** was co-evaporated with pyridine and 1,4-dioxane, and then, a solution of **9** in MeCN was added. *^e^* A mixture of **9**, **10** and **11** was co-evaporated with pyridine and 1,4-dioxane and treated with promoters. *^f^* Glycosylation reactions were carried out in the presence of 1.5 equivalents of **9**, 1.8 equivalents of *p*-TolSCl and 3.6 equivalents of AgOTf against **10** as followed by acetylation with Ac_2_O (*ca.* 10 equivalents) and DMAP (catalytic amount).

**Table 2:**
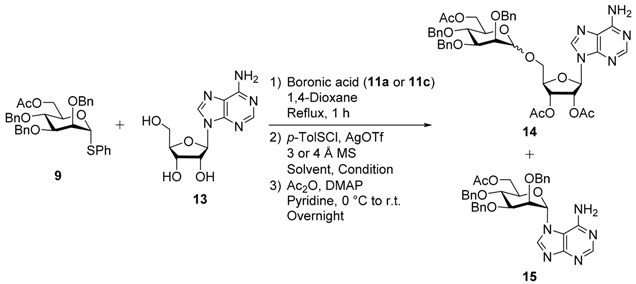
*O*-glycosylation of adenosine **13** with thiomannoside **9** in the absence and presence of boronic acid.

Entry *^a^*	Boronic Acid *^b^*	Solvent	Condition	Yield of 14 (for 3 Steps) *^c^*	Yield of 15 (for 3 Steps)
1	-	MeCN	−20 °C, 1.5 h	<10% (complex mixture)	not isolated
2 *^d^*	PhB(OH)_2_ (**11a**)	MeCN	−20 °C, 1.5 h	14% (α/β = 1/1.0)	6%
3 *^d^*	4-CF_3_PhB(OH)_2_ (**11c**)	EtCN	−40 °C, 1.5 h	11% (α/β = 1/1.2)	27%

*^a^* Glycosylation reactions were carried out in the presence of 1.5 equivalents of **9**, 3.0 equivalents of *p*-TolSCl and 6.0 equivalents of AgOTf against **13**. Acetylation reactions were carried out in the presence of *ca.* 10 equivalents of Ac_2_O and the catalytic amount of DMAP. *^b^* Stoichiometry of **11** was 1.5 equivalents against **13**. *^c^* The α/β ratio was determined by ^1^H NMR. *^d^* A mixture of **9**, **13** and **11** was co-evaporated with pyridine and 1,4-dioxane and treated with promoters.

**Table 3:**
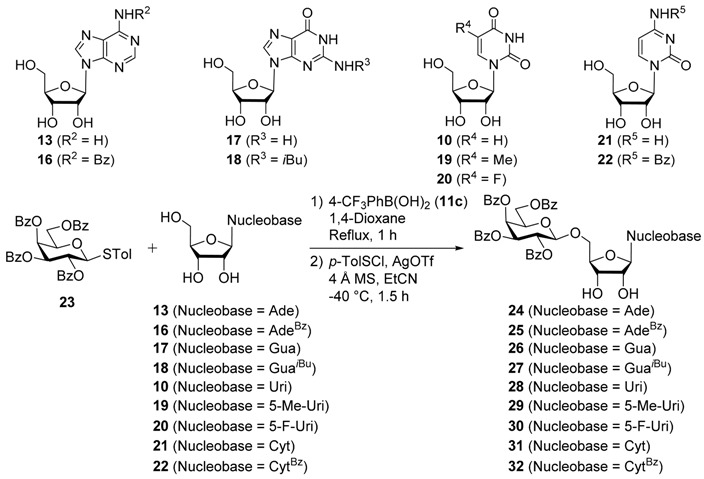
*O*-glycosylation of nucleosides **10**, **13** and **16**–**22** with the thiogalactoside **23**.

Entry *^a^*	Acceptor	Product	Yield (for 2 Steps)
1	**13** (Ade)	β-**24**	42%
2	**16** (Ade^Bz^)	β-**25**	30%
3	**17** (Gua)	β-**26**	12%
4	**18** (Gua*^i^*^Bu^)	β-**27**	44%
5	**10** (Uri)	β-**28**	42% (*ca.* 15%: nucleobase = 5-STol-Uri)
6	**19** (5-Me-Uri)	β-**29**	53%
7	**20** (5-F-Uri)	β-**30**	61%
8	**21** (Cyt)	β-**31**	55%
9	**22** (Cyt^Bz^)	β-**32**	40%

*^a^* Glycosylation reactions were carried out in the presence of 1.5 equivalents of **23**, 3.0 equivalents of *p*-TolSCl and 6.0 equivalents of AgOTf against the acceptor (**10**, **13** or **16**–**22**). Stoichiometry of **11c** was 1.5 equivalents against acceptor (**10**, **13** or **16**–**22**). A mixture of **23**, acceptor (**10**, **13**, or **16**–**22**) and **11c** was co-evaporated with pyridine and 1,4-dioxane and treated with promoters.

**Table 4:**
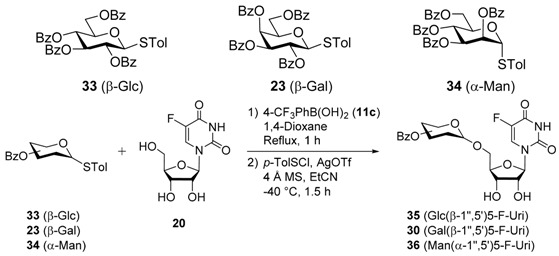
*O*-Glycosylation of 5-fluorouridine **20** with thioglycosides **23**, **33** and **34**.

Entry *^a^*	Donor	Product	Yield (for 2 Steps)
1	**33** (β-Glc)	β-**35**	54%
2 *^b^*	**23** (β-Gal)	β-**30**	61%
3	**34** (α-Man)	α-**36**	<39% (mixture)

*^a^* Glycosylation reactions were carried out in the presence of 1.5 equivalents of donor (**23**, **33** or **34**), 3.0 equivalents of *p*-TolSCl and 6.0 equivalents of AgOTf against **20**. Stoichiometry of **11c** was 1.5 equivalents against **20**. A mixture of donor (**23**, **33** or **34**), **20** and **11c** was co-evaporated with pyridine and 1,4-dioxane and treated with promoters. *^b^* Taken from Entry 7 of Table 3 in this manuscript.

**Table 5:**
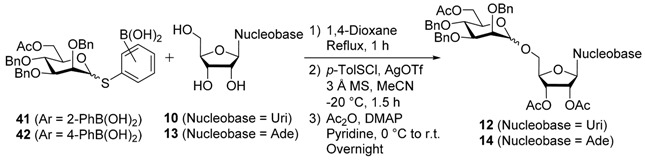
*O*-glycosylation of uridine **10** and adenosine **13** with thioglycosides **41** and **42**.

Entry *^a^*	Donor	Acceptor	Product	Yield (for 3 Steps) *^b^*
1	**41** (Ar = 2-PhB(OH)_2_) (α form)	**10** (Nucleobase = Uri)	**12**	44% (α/β = 1.9/1)
2	**41** (Ar = 2-PhB(OH)_2_) (α form)	**13** (Nucleobase = Ade)	**14**	16% (α/β = 1.3/1)
3	**42** (Ar = 4-PhB(OH)_2_) (α/β = 1/1.0)	**10** (Nucleobase = Uri)	**12**	36% (α/β = 2.1/1)
4	**42** (Ar = 4-PhB(OH)_2_) (α/β = 1/1.0)	**13** (Nucleobase = Ade)	**14**	14% (α/β = 1.1/1)

*^a^* Glycosylation reactions were carried out in the presence of 1.5 equivalents of donor (**41** or **42**), 3.0 equivalents of *p*-TolSCl and 6.0 equivalents of AgOTf against the acceptor (**10** or **13**). A mixture of donor (**41** or **42**) and acceptor (**10** or **13**) was co-evaporated with pyridine and 1,4-dioxane and treated with promoters. Acetylation reactions were carried out in the presence of *ca.* 10 equivalents of Ac_2_O and a catalytic amount of DMAP. *^b^* The α/β ratio was determined by ^1^H NMR.

## Supplementary Materials

Supplementary Materials are available online.
