# Supplementary material for: Synthesis of Disaccharide Nucleosides Utilizing the Temporary Protection of the 2′,3′-cis-Diol of Ribonucleosides by a Boronic Ester
Source: Molecules. 2017 Oct 1;22(10):1650. doi: 10.3390/molecules22101650 (PMC6151833; doi:10.3390/molecules22101650)
Supplement: Supplementary file 1 [file molecules-22-01650-s001.pdf]

Supplementary Materials

Synthesis of Disaccharide Nucleosides Utilizing the Temporary Protection of the 2',3'-*cis*-Diol of Ribonucleosides by a Boronic Ester

Hidehisa Someya [1], Taiki Itoh [1] and Shin Aoki [1,2,*]

[1] Faculty of Pharmaceutical Sciences, Tokyo University of Science,

2641 Yamazaki, Noda, Chiba 278-8510, Japan,

[2] Imaging Frontier Center, Tokyo University of Science,

2641 Yamazaki, Noda, Chiba 278-8510, Japan,

*Correspondence: E-mail, shinaoki@rs.noda.tus.ac.jp; Tel.: (+81) 4-7121-3670

1. Preparation of Authentic Samples and Glycosyl Donors

1.1. Synthesis of Disaccharide Nucleosides Containing 1”,5’-Glycosidic Linkage

We prepared the disaccharide nucleosides containing 1”,5’-glycosidic linkage as authentic samples. Disaccharide nucleoside including the uracil moiety **12** was prepared by the *O*-glycosylation of **50** [1] with **9** in the presence of *p*-TolSCl, AgOTf, and 2,6-di-tert-butyl-4-methylpyridine (DTBMP) [2] followed by the cleavage of acetonide protecting group and acetylation (Scheme S1a). In addition, disaccharide nucleoside possessing the adenine moiety **14** was synthesized from **51** [3–5] and **9** in a similar manner (Scheme S1b).

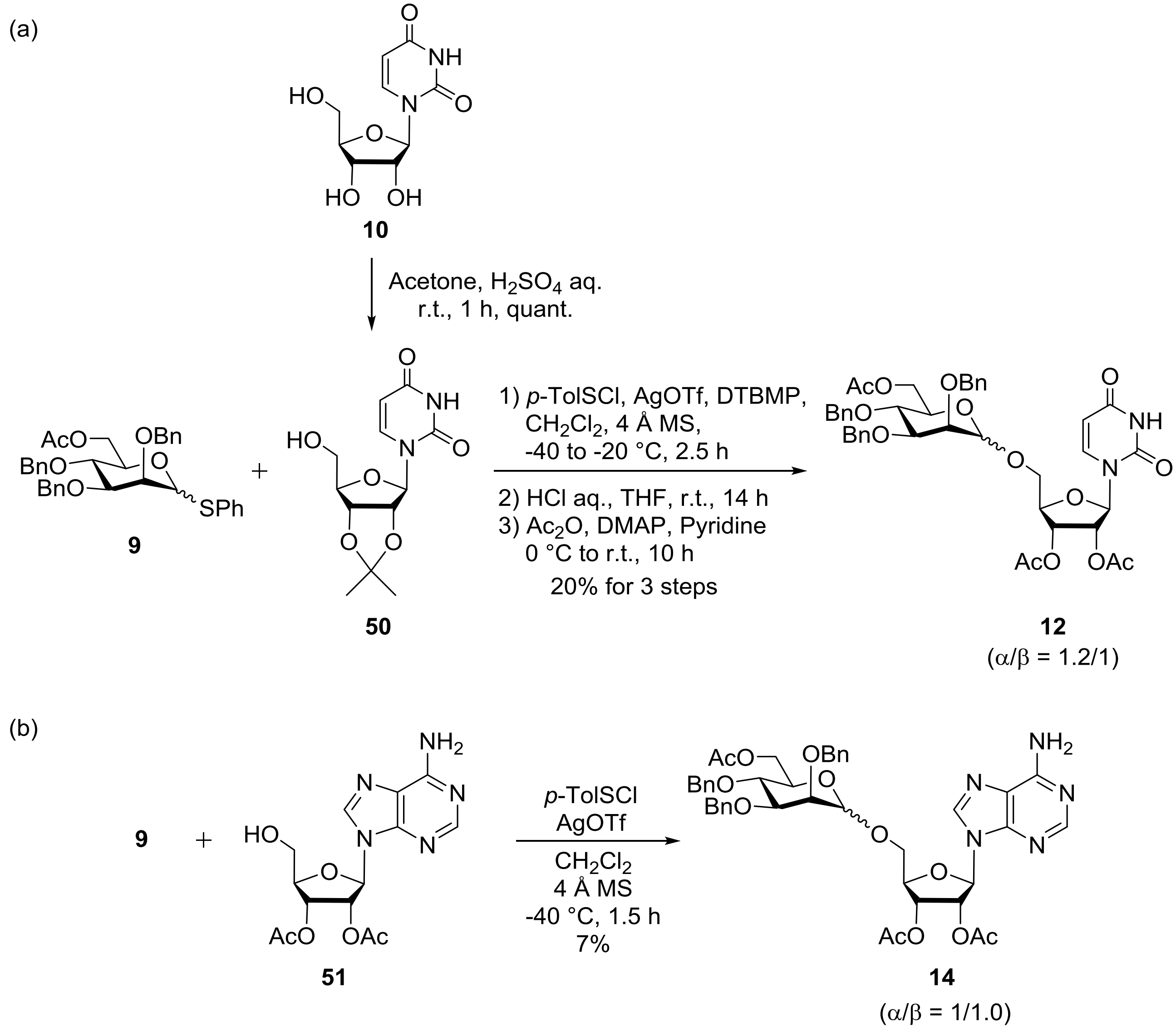

Scheme S1. Synthesis of disaccharide nucleosides as authentic samples.

1.2. Synthesis of Glycosyl Donors Containing Boronic Acid on Leaving Group

For the synthesis of glycosyl donors containing a boronic acid on the leaving group, the 2-mercaptophenylboronic acid pinacol ester **53** was synthesized from thiophenol **52** using *n*-BuLi-TMEDA complex and trimethyl borate followed by the protection of the boronic acid with pinacol (Scheme S2) [6–7].

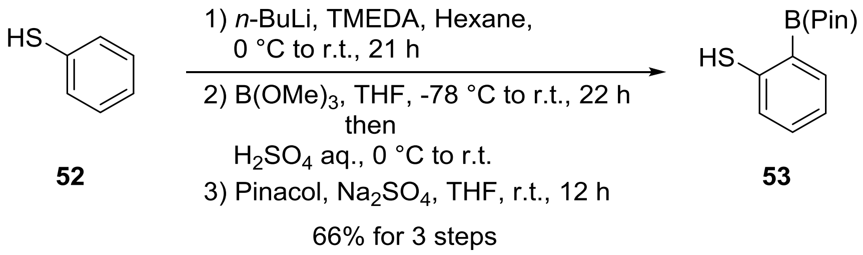

Scheme S2. Synthesis of 2-mercaptophenylboronic acid pinacol ester **53**.

The *S*-glycosylation of **54** [8] with **53** in the presence of BF_3•OEt_2 gave thioglycoside **55**, in which the pinacol group was converted to **41** via **56** as shown in Scheme S3 [9]. The thioglycoside **42** containing a boronic acid on the 4 (*para*) position was also prepared by the reaction of 4-mercaptophenylboronic acid with **54**.

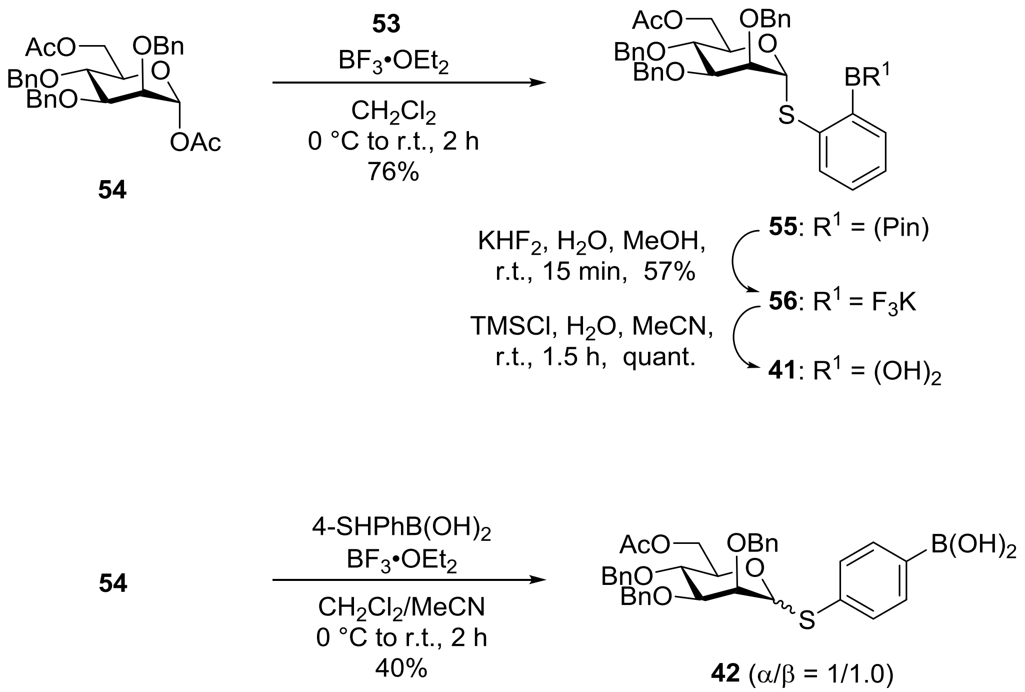

Scheme S3. Synthesis of glycosyl donors containing a boronic acid on the leaving group.

2. Materials and Methods

2',3'-Di-O-acetyl-5'-O-(6"-O-acetyl-2",3",4"-tri-O-benzyl-α/β-D-mannopyranosyl)uridine (**12**) (Scheme S1a): A mixture of **9** (30.0 mg, 51.3 μmol), **50** (29.2 mg, 102 μmol) and activated 4 Å molecular sieves (150 mg) was stirred in anhydrous CH_2Cl_2 (1.5 mL) for 30 min, then cooled to -40 °C, to which DTBMP (31.6 mg, 154 μmol), AgOTf (39.5 mg, 154 μmol) and *p*-TolSCl (17.0 μL, 129 μmol) were added at the same temperature. The reaction mixture was stirred at the same temperature for 1 hr and then allowed to warm to -20 °C. After stirring for 1.5 hr, the reaction mixture was quenched with saturated aqueous $NaHCO_3$, diluted with $CHCl_3$, and filtered through Celite. The organic layer was washed with saturated aqueous $NaHCO_3$ and brine, dried over Na_2SO_4, filtered, and concentrated under reduced pressure. The residue was purified by silica gel column chromatography ($CHCl_3$/MeOH = 1/0 to 90/1) followed by GPC ($CHCl_3$) to give the product as a colorless syrup (12.0 mg). To a solution of resulting compound in THF (300 μL), 2M aqueous HCl (300 μL) was added at room temperature. After stirring at the same temperature for 14 hr, the reaction mixture was neutralized with saturated aqueous $NaHCO_3$,

extracted with $CHCl_3$, washed with brine, dried over Na_2SO_4, filtered, and concentrated under reduced pressure. The residue was purified by silica gel column chromatography ($CHCl_3$/MeOH = 1/0 to 70/1) to give a product (7.7 mg). To a solution of this product in pyridine (200 μL), Ac_2O (13.8 μL, 146 μmol) and DMAP (catalytic amount) were added at 0 °C. The reaction mixture was stirred at the same temperature for 30 min and allowed to warm to room temperature. After stirring for 10 hr, the reaction mixrture was diluted with $CHCl_3$, washed with 1M aqueous HCl, saturated aqueous $NaHCO_3$, and brine, dried over Na_2SO_4, filtered, and concentrated under reduced pressure. The residue was purified by silica gel column chromatography ($CHCl_3$/MeOH = 1/0 to 90/1) to give **12** as a colorless amorphous solid (8.1 mg, 20% yield for 3 steps, α/β = 1.2/1).

2',3'-Di-O-acetyl-5'-O-(6"-O-acetyl-2",3",4"-tri-O-benzyl-α/β-D-mannopyranosyl)adenosine (**14**) (Scheme S1b): A mixture of **9** (83.2 mg, 142 μmol), **51** (100 mg, 285 μmol) was stirred with activated 4 Å molecular sieves (300 mg) in anhydrous CH_2Cl_2 (3.0 mL) for 30 min, then cooled to -40 °C, to which AgOTf (110 mg, 428 μmol) and *p*-TolSCl (37.6 μL, 284 μmol) were added at the same temperature. After stirring for 1.5 hr, the reaction mixture was quenched with saturated aqueous $NaHCO_3$, diluted with $CHCl_3$, and filtered through Celite. The organic layer was washed with saturated aqueous $NaHCO_3$ and brine, dried over Na_2SO_4, filtered, and concentrated under reduced pressure. The residue was purified by silica gel column chromatography ($CHCl_3$/MeOH = 1/0 to 60/1) followed by GPC ($CHCl_3$) to give a **14** as a colorless syrup (8.1 mg, 7% yield, α/β = 1/1.0).

2- Mercaptophenylboronic acid pinacol ester (**53**): A mixture of thiophenol **52** (750 μL, 7.33 mmol) and TMEDA (2.4 mL, 16.1 mmol) were stirred at 0 °C, to which 1.6M *n*-BuLi in hexane (13.8 mL, 27.0 mmol) was added at the same temperature. The reaction mixture was stirred for 3 hr at 0 °C and was allowed to warm to room temperature. After stirring for 18 hr, the reaction mixture became a white suspension. The white precipitate was collected by centrifugation, washed with anhydrous hexanes and

dissolved in anhydrous THF (7.0 mL). The solution was cooled to -78 °C, to which $B(OMe)_3$ (1.1 mL, 9.53 mmol) was added. The reaction mixture was stirred at the same temperature for 3 hr and then allowed to warm to room temperature. After stirring for 19 hr, the reaction mixture was quenched with 10% aqueous H_2SO_4 in an ice bath and extracted with $CHCl_3$, washed with brine, dried over Na_2SO_4, filtered, and concentrated under reduced pressure. The residue was dissolved in anhydrous THF (20.0 mL) at room temperture, to which Na_2SO_4 (3.00 g, 21.1 mmol) and pinacol (1.30 g, 11.0 mmol) were added at the same temperature. The reaction mixture was stirred at the same temperature. After stirring for 12 hr, the reaction mixture was filtered and concentrated under reduce pressure, the residue was diluted with $CHCl_3$, washed with H_2O, and brine, dried over Na_2SO_4, filtered, and concentrated under reduced pressure. The residue was purified by silica gel column chromatography (hexanes/AcOEt = 100/1) to give **53** as a colorless liquid (1.18 g, 68% yield for 3 steps): ^{1}H NMR (300 MHz, $CDCl_3$, TMS): δ= 7.75 (dd, J = 7.2, 1.2 Hz, 1H), 7.25 (td, J = 6.8, 1.6 Hz, 1H), 7.22 (dd, J = 7.2, 1.2 Hz, 1H), 7.08 (td, J = 7.2, 1.6 Hz, 1H), 5.20 (s, 1H), 1.37 (s, 12H) ppm; ^{13}C NMR (100 MHz, $CDCl_3$, TMS): δ= 140.5, 137.2, 131.3, 128.5, 123.9, 84.2, 24.9 ppm; ^{11}B NMR (128 MHz, $CDCl_3$, BF_3•OEt_2): δ= 26.13 (brs) ppm; IR (ATR): ν = 3063, 2979, 2932, 2569, 1589, 1559, 1476, 1427, 1381, 1373, 1343, 1315, 1266, 1214, 1141, 1127, 1102, 1049, 1038, 963, 856, 830, 755, 735, 709, 670, 654, 580 cm^{-1}; HRMS (EI+): calcd for $[M]^+$, $C_{12}H_{17}{}^{10}BO_2S$, 235.1079; found, 235.1083.

2-(4,4,5,5-Tetramethyl-1,3,2-dioxaborolan-2-yl)phenyl 6-O-acetyl-2,3,4-tri-O-benzyl-1-thio-α-D-mannopyranoside (**55**): To a solution of **54** (1.10 g, 2.06 mmol) and **53** (729 mg, 3.09 mmol) in anhydrous CH_2Cl_2 (21.0 mL), BF_3•OEt_2 (776 μL, 6.18 mmol) was added at -0 °C. The reaction mixture was stirred at the same temperature for 30 min and allowed to warm to room temperature. After stirring for 2 hr, the reaction mixture was quenched with saturated aqueous $NaHCO_3$ and diluted with AcOEt. The organic layer was washed with saturated aqueous $NaHCO_3$ and brine, dried over Na_2SO_4, filtered, and concentrated under reduced pressure. The residue was purified by silica gel column chromatography

(hexanses/AcOEt = 20/1 to 8/1) to give **55** as a colorless syrup (1.11 g, 76% yield): ^{1}H NMR (300 MHz, $CDCl_3$, TMS): δ = 7.62 (dd, J = 7.5, 1.8 Hz, 1H), 7.50 (d, J = 7.2 Hz, 1H), 7.40-7.21 (m, 17H), 5.74 (d, J = 1.5 Hz, 1H), 4.95 (d, J = 11.1 Hz, 1H), 4.74 (d, J = 12.0 Hz, 1H), 4.68-4.56 (m, 4H), 4.41-4.24 (m, 3H), 4.05-3.94 (m, 3H), 2.00 (s, 3H), 1.33 (s, 12H) ppm; ^{13}C NMR (100 MHz, $CDCl_3$, TMS): δ = 170.8, 139.0, 138.2, 138.1, 138.1, 135.3, 132.2, 130.9, 128.4, 128.4, 128.3, 128.0, 127.8, 127.7, 127.5, 126.7, 85.2, 84.1, 80.2, 76.6, 75.2, 74.7, 72.0, 71.7, 70.9, 63.5, 24.9, 24.7, 20.8 ppm; ^{11}B NMR (128 MHz, $CDCl_3$, BF_3•OEt_2): δ = 26.34 (brs) ppm; IR (ATR): ν = 3063, 3031, 2978, 2932, 2869, 1739, 1586, 1497, 1455, 1429, 1380, 1371, 1348, 1315, 1238, 1144, 1101, 1043, 1028, 962, 910, 857, 834, 735, 696, 669, 658, 604, 580 cm^{-1}; HRMS (FAB+): calcd for $[M+Na]^+$, $C_{41}H_{47}{}^{10}BO_8SNa$, 732.3019; found, 732.3018; $[\alpha]^{23}_D$ = +84.6 (c = 1.0, $CHCl_3$).

Potassium [2-(6-O-Acetyl-2,3,4-tri-O-benzyl-α-D-mannopyranosyl)thiophenyl] trifluoroborate (**56**): To a solution of **55** (165 mg, 0.232 mmol) in MeOH (1.0 mL), 7.0 M aqueous KHF_2 (331 μL, 2.32 mmol) was added at room temperature. After stirring at the same temperature for 15 min, the reaction mixture was concentrated under reduced pressure, diluted with hot acetone and filtered through Celite. The solution was concentrated under reduced pressure. The residue was purified by silica gel column chromatography ($CHCl_3$/acetone = 5/1 to 1/1) to give **56** as a colorless syrup (91.3 mg, 57% yield): ^{1}H NMR (300 MHz, acetone-d_6, TMS): δ = 7.64 (dd, J = 6.6, 1.8 Hz, 1H), 7.46-7.24 (m, 16H), 7.11-7.01 (m,2H), 5.84 (s, 1H), 4.95 (d, J = 11.4 Hz, 1H), 4.76 (d, J = 12.3 Hz, 1H), 4.71-4.60 (m, 3H), 4.57 (d, J = 11.7Hz, 1H), 4.45-4.36 (m, 1H), 4.33-4.22 (m, 3H), 3.96 (dd, J = 9.3, 3.0 Hz, 1H), 3.91 (d, J = 9.6 Hz, 1H), 1.98 (s, 3H) ppm; ^{13}C NMR (100 MHz, aceton-d_6, TMS): δ = 170.9, 139.6, 139.6, 139.5, 138.0, 133.4, 128.9, 128.9, 128.8, 128.8, 128.6, 128.6, 128.5, 128.4, 128.4, 128.2, 128.1, 128.0, 127.9, 127.3, 126.7, 86.1, 81.2, 77.2, 75.6, 75.2, 71.9, 71.5, 71.4, 64.1, 55.2, 31.9, 20.7 ppm; ^{11}B NMR (128 MHz, acetone-d_6, BF_3•OEt_2): δ = 4.20 (brs) ppm; ^{19}F NMR (376 MHz, aceton-d_6, TFA): δ = -140.99 (s) ppm; IR (ATR): ν = 3475, 3032, 2873, 1737, 1585, 1559, 1497, 1455, 1428, 1367, 1238, 1191, 1091, 1073,

1023, 947, 735, 696, 671, 606 cm^{-1}; HRMS (ESI): calcd for $[M-K]^-$, $C_{35}H_{35}{}^{10}BO_6F_3S^-$, 650.2241; found, 650.2238; $[\alpha]^{22}_D$ = +72.3 (c = 1.0, acetone).

2-Boronophenyl 6-O-acetyl-2,3,4-tri-O-benzyl-1-thio-α-D-mannopyranoside (**41**): To a solution of **56** (105 mg, 0.152 mmol) in MeCN (1.5 mL), H_2O (10.9 μL, 0.605 mmol) and TMSCl (76.8 μL, 0.605 mmol) were added at room temperature. After stirring at the same temperature for 1.5 hr, the reaction mixture was diluted with H_2O, extracted with $CHCl_3$, washed brine, dried over Na_2SO_4, filtered, and concentrated under reduced pressure. The residue was purified by silica gel column chromatography (hexanes/AcOEt = 3/1) to give **41** as a colorless syrup (95.1 mg, 99%.): ^{1}H NMR (300 MHz, $CDCl_3$, TMS): δ = 8.10-7.76 (m, 1H), 7.46-7.27 (m, 18H), 5.82 (s, 2H), 5.33 (d, J = 1.8 Hz, 1H), 4.92 (d, J = 11.1 Hz, 1H), 4.69-4.52 (m, 5H), 4.33 (d, J = 3.6 Hz, 2H), 4.27-4.18 (m, 1H), 3.98-3.81 (m, 3H), 2.03 (s, 3H) ppm; ^{13}C NMR (100 MHz, $CDCl_3$, TMS): δ = 170.7, 137.9, 137.7, 137.4, 137.0, 136.4, 135.5, 130.8, 128.4, 128.4, 128.4, 128.0, 127.9, 127.9, 127.8, 87.9, 79.4, 75.9, 75.1, 74.3, 72.0, 71.8, 71.8, 63.2, 58.7, 20.7, 17.1 ppm; ^{11}B NMR (128 MHz, $CDCl_3$, BF_3•OEt_2): δ = 29.53 (brs) ppm; IR (ATR): ν = 3414, 3064, 3031, 2871, 1740, 1585, 1559, 1497, 1455, 1432, 1368, 1338, 1313, 1239, 1090, 1026, 910, 866, 846, 738, 696, 665, 646, 605 cm^{-1}; HRMS (ESI): calcd for $[M+Cl]^-$, $C_{35}H_{37}{}^{10}BO_8S^{35}Cl^-$, 662.2033; found, 662.2031; $[\alpha]^{24}_D$ = +84.5 (c = 1.0, $CHCl_3$).

4-Boronophenyl 6-O-acetyl-2,3,4-tri-O-benzyl-1-thio-α/β-D-mannopyranoside (**42**): *S*-Glycosylation using **54** (400 mg, 0.748 mmol), 4-mercaptophenylboronic acid (231 mg, 1.50 mmol), BF_3•OEt_2 (500 μL, 3.74 mmol), anhydrous CH_2Cl_2 (4.5 mL), and anhydrous MeCN (3.0 mL) was conducted according to the procedure used for the synthesis of **55**. The residue was purified by silica gel column chromatography (hexanses/AcOEt = 4/1 to 2/1) to give **42** as a colorless syrup (186.6 mg, 40% yield, α/β = 1/1.0): ^{1}H NMR (300 MHz, $CDCl_3$, TMS): δ = 8.09 (d, J = 8.1 Hz, 1H), 7.65 (d, J = 8.1 Hz, 0.5H), 7.54 (d, J = 7.8 Hz, 1H), 7.44 (d, J = 8.1 Hz, 0.5H), 7.68-7.27 (m, 16H), 5.73 (d, J = 0.9 Hz, 0.5H), 5.65 (d, J = 1.5 Hz,

0.5H), 4.95 (dd, J = 11.1, 3.6 Hz, 1H), 4.80-4.54 (m, 6H), 4.45-4.21 (m, 4H), 4.00 (m, 2H), 3.88 (td, J = 9.0, 3.0 Hz, 1H), 2.04 (s, 1.5H), 2.02 (s, 1.5H) ppm; ^{13}C NMR (75 MHz, $CDCl_3$, TMS): δ = 171.0, 170.9, 140.2, 138.0, 137.9, 137.7, 137.1, 136.0, 134.4, 133.9, 130.0, 130.0, 129.4, 128.8, 128.5, 128.5, 128.4, 128.2, 128.2, 128.1, 127.9, 127.9, 127.9, 127.8, 127.6, 85.0, 84.7, 80.2, 80.1, 77.2, 76.1, 76.0, 75.2, 74.5, 74.4, 72.2, 72.1, 72.0, 71.2, 71.0, 63.4, 63.3, 58.8, 58.5, 23.3, 20.9, 20.9, 18.4, 17.2 ppm; ^{11}B NMR (128 MHz, $CDCl_3$, BF_3•OEt_2): δ = 29.75 (brs) ppm; IR (ATR): ν = 3443, 3065, 3031, 2871, 1739, 1658, 1593, 1548, 1497, 1455, 1396, 1365, 1342, 1317, 1240, 1087, 1041, 1016, 910, 829, 734, 696, 666, 645, 630, 604 cm^{-1}; HRMS (ESI): calcd for $[M+Cl]^-$, $C_{35}H_{37}{}^{10}BO_8S^{35}Cl^-$, 662.2033; found, 662.2036.

References

1. Fujihashi, M.; Ishida, T.; Kuroda, S.; Kotra, L.P.; Pai, E.F.; Miki, K. Substrate distortion contributes to the catalysis of orotidine 5'-monophosphate decarboxylase. *J. Am. Chem. Soc.* **2013**, *135*, 17432–17443.

2. Crich, D.; Sun, S. Direct formation of β-mannopyranosides and other hindered glycosides from thioglycosides. *J. Am. Chem. Soc.* **1998**, *120*, 435–436.

3. Beaton, G.; Jones, A.S.; Walker, R.T. The chemistry of 2',3'-seconucleosides III. Synthesis and reactions of purine-2',3'-secoribonucleosides. *Tetrahedron* **1988**, *44*, 6419–6428.

4. Lavergne, T.; Baraguey, C.; Dupouy, C.; Parey, N.; Wuensche, W.; Sczakiel, G.; Vasseur, J.J.; Debart, F. Synthesis and preliminary evaluation of pro-RNA 2'-*O*-masked with biolabile pivaloyloxymethyl groups in an RNA interference assay. *J. Org. Chem.* **2011**, *76*, 5719–5731.

5. Tonn, V.C.; Meier, C. Solid-phase synthesis of (poly)phosphorylated nucleosides and conjugates. *Chem. Eur. J.* **2011**, *17*, 9832–9842.

6. Smith, K.; Lindsay, C.M.; Pritchard, G.J. Directed lithiation of arenethiols. *J. Am. Chem. Soc.* **1989**, *111*, 665–669.

7. Sakamaki, S.; Kawanishi, E.; Nomura, S.; Ishikawa, T. Aryl-β-*C*-glucosidation using glucal boronate: application to the synthesis of tri-*O*-methylnorbergenin. *Tetrahedron* **2012**, *68*, 5744–5753.

8. Tennant-Eyles, R.J.; Davis, B.G.; Fairbanks, A.J. Peptide templated glycosylation reactions. *Tetrahedron: Asymmetry* **2000**, *11*, 231–243.

9. Yuena, A.K.L.; Hutton, C.A. Deprotection of pinacolyl boronate esters via hydrolysis of intermediate potassium trifluoroborates. *Tetrahedron Lett.* **2005**, *46*, 7899–7903.
